# Inflorescences of *Cuscuta* (Convolvulaceae): Diversity, evolution and relationships with breeding systems and fruit dehiscence modes

**DOI:** 10.1371/journal.pone.0286100

**Published:** 2023-05-19

**Authors:** Morgan Glofcheskie, Tristan Long, Anna Ho, Mihai Costea

**Affiliations:** Department of Biology, Wilfrid Laurier University, Waterloo, Ontario, Canada; Hazara University, PAKISTAN

## Abstract

*Cuscuta* (dodder) includes ca. 200 species of plant obligate stem parasites with enormous ecological and economical significance. Inflorescences have been historically used in *Cuscuta* for species descriptions and identification keys, but no comprehensive study exists to date. The main objectives of this study were to survey the diversity and evolution of inflorescences and to uncover their possible form-function relationships. The inflorescence architecture of 132 *Cuscuta* taxa was analysed using herbarium specimens and eight species were grown to study their inflorescence development. Inflorescence traits were mapped into a genus phylogeny obtained from a combined analysis of nuclear ITS and plastid trnL-F sequences. To test the hypothesis that inflorescence architecture is connected to sexual reproduction, correlations between inflorescence traits (using Principal Components), sexual reproductive traits (pollen/ovule ratios, corolla length and diameter), fruit charaters (fruit length and width), and the modes of dehiscence were analyzed. Based on their development, three major types of inflorescences were observed: “*Cuscuta type*”, a simple, monochasial scorpioid cyme; “*Monogynella type*”, a compound monochasial scorpioid cymes with the longest primary axes having prolonged vegetative growth and giving the appearance of thyrses; and “*Grammica type*”, a compound monochasial scorpiod cymes with up to five orders of axes. Maximum likelihood analyses suggested *Monogynella* as the ancestral type, while *Cuscuta* and *Grammica* were derived. Overall, the total length of axes exhibited a reduction trend throughout the genus evolution, but it was not correlated with the pedicels length. Inflorescences with similar architectures may exhibit contrasting pollen-ovule ratios. Positive significant correlations were noted between the size of the flower traits and pollen-ovule ratios. Several modes of dehiscence had statistically significant different total axes lengths, suggesting that the infructescence architecture is connected to the modes of dehiscence in *Cuscuta* and therefore seed dispersal.

## Introduction

*Cuscuta* (dodders) includes ca. 200 species of obligate stem parasitic plants with worldwide distribution [1–4]. Dodders inhabit a great variety of natural ecosystems, from temperate to tropical, desert to wetland, littoral to high mountains, grasslands, forests, saline and disturbed, in which they act as keystone species and ecosystem engineers [4–6]. *Cuscuta* species can also be economically detrimental as infestation by some 15–20 species worldwide can result in major yield losses in numerous agricultural and horticultural crops [7–10]. Native *Cuscuta* species may require conservation [e.g., 11], while weedy/invasive species demand strict control [7–10].

The evolution to parasitism in *Cuscuta* has led to major reductions of their vegetative organs (roots, stems and leaves) and a diversification of their reproductive organs [12–15]. These reductions have historically limited the number of characters available for species delimitation primarily to the flowers, and to a less extent, fruits [1–3]. During the last decade, efforts have been made to explore the diversity and evolution of other characters such as the infrastaminal scales [16], pollen [17], multicellular protuberances with stomata on flowers and stems [18], fruits [19], and seeds [20].

Inflorescence architecture in angiosperms varies considerably at different taxonomic levels (e.g., family, genus and species [21–26]) and inflorescence classification and terminology have been inconsistent and conflicting (e.g., discussed by [26–29]). In *Cuscuta*, Mirande [30] studied the inflorescence of eight species and concluded that their fundamental units are uniparous scorpioid cymes. Although commonly referred to as “cymose” subsequently (e.g., [2, 3, 31, 32], Mirande’s study [30] has been largely overlooked and has remained uncorroborated. The terminology used for *Cuscuta* inflorescences in species descriptions and identification keys has employed racemose analogies (e.g., spiciform, paniculiform, corymbiform, umbelliform; e.g., [2, 3, 33–48] and no genus-wide study has been conducted to date. Similarly, the diversity and evolution of inflorescences have not been studied in Convolvulaceae, more broadly.

Inflorescences have a direct effect on plant reproductive success as they connect the vegetative stages within a plant’s life cycle with sexual reproduction, allowing for flower displays, effective pollen transfer and fertilization [49–53]. Inflorescences are spatially dynamic and their architectural temporal patterns prolong sexual receptivity and increase reproductive assurance [e.g., 29, 50]. Not surprisingly, in some plants, the specific arrangement of axes and sequence of flower development was found to be correlated with the breeding systems [54–56]. *Cuscuta* species possess a wide range of mixed-mating breeding systems that range from obligate xenogamous to facultative autogamous [15], and thus they represent an ideal group to test for a possible relationship between the inflorescence architecture and breeding systems.

Anecdotal observations made in *Cuscuta* subg. *Grammica* suggested that inflorescences of species with indehiscent fruits are denser, glomerulate, whereas species with regularly circumscissile capsules usually possess lax inflorescences [19, 45]. Ho and Costea [19] reported that in addition to the indehiscent (IN) and dehiscent (DE) fruits, two irregular modes of dehiscence exist in *Cuscuta*. Irregular dehiscence type A (IrA) is caused by a thin pericarp at the base of the capsules which break irregularly in this area as the seeds develop. Irregular dehiscence type B fruits (IrB) are structurally indistinguishable from indehiscent ones, but a small percentage of fruits break irregularly because of the pressures created within dense infructescences. Modes of dehiscence in *Cuscuta* are important because they affect the seed dispersal and germination [19], which in turn may influence both population level evolutionary processes (e.g., [57, 58]), as well as species level patterns of distribution and diversification (e.g., [19, 58–60]). However, a potential relationship between the inflorescence architecture and the modes of dehiscence remains to be established in dodders.

The objectives of this study are: 1) Survey the morphological diversity of inflorescences in *Cuscuta* and reconstruct ancestral character states; discuss the usefulness of inflorescences for the systematics and taxonomy of dodders, as well as consider these results in the broader context of Convolvulaceae; (2) Investigate possible relationships between the inflorescence architecture and sexual reproductive traits such as pollen-ovule ratios as a proxy for breeding systems, flower diameter/length, fruit width/length, and the modes of fruit dehiscence in *Cuscuta*.

## Materials and methods

### Taxonomic sampling and plant materials used

Inflorescence architecture was comparatively studied in 132 *Cuscuta* taxa using herbarium specimens (S1 Appendix). Representatives of all four subgenera, *Cuscuta*, *Monogynella*, *Pachystigma*, and *Grammica* were included. Three to nine herbarium specimens were examined per taxon (S1 Appendix). Specimens from the following herbaria were annotated and sampled: ALTA, ARIZ, ASU, B, BRIT, CANB, CAS, CHSC, CIIDIR, CIMI, CTES, DAO, F, G, GH, IEB, IND, JEPS, K, LIL, MEL, MEXU, MICH, MO, MT, MTMG, NBG, NFLD, NMC, NY, OKLA, OSC, P, PACA, PERTH, QCNE, QFA, QUE, RB, RSA, S, SASK, SD, SGO, SI, SMU, TEX, TRTE, UBC, UCR, UC, UNB, UNM, UPRRP, US, VEN, XAL, WLU (abbreviations from Index Herbarium [61]). Five inflorescence units were removed from each herbarium specimen. Inflorescences were rehydrated in warm 50% ethanol, detangled, and imaged under a Nikon SMZ1500 stereo microscope using a PaxCam Arc digital camera and Pax-it! 2 version 1.5.1.0 imaging software (MIS Inc, Villa Park, IL).

In addition, eight *Cuscuta* species were grown in the greenhouse at Wilfrid Laurier University during the summer of 2021 to observe the development of their inflorescences: *C*. *epithymum* (Subg. *Cuscuta*); *C*. *monogyna* and *C*. *lupuliformis* (Subg. *Monogynella*); *C*. *costaricensis*, *C*. *gracillima*, *C*. *chapalana*, *C*. *californica* var. *californica* and *C*. *campestris* (Subg. *Grammica*). Seeds were scarified with a 99.99% sulfuric acid treatment for 30 minutes [62, 63]. After the treatment, seeds were transferred to 140 mm sterile Petri dishes containing two Whatman filter paper rings that were moistened with Milli-Q water. Germination took place in an incubator at 32°C, under light (150 μmol m^-2^ s^-1^). *Plectranthus scutellarioides* commercial plants were used as hosts for all *Cuscuta* species [64, 65] except for *C*. *epithymum* for which we used *Medicago sativa* (alfalfa) [10]. Once *Cuscuta* seeds germinated and seedlings were ca. 2–3 cm long, they were individually immersed with their root organ in a 0.5 mL microcentrifuge tube filled with Milli-Q water to prevent desiccation and prolong seedling survival [66]. Microcentrifuge tubes were placed in the soil at the base of the young host plants or attached to their shoots to facilitate seedling attachment.

Inflorescences of different ages of another species of subg. *Grammica*—*C*. *gronovii* var. *gronovii*—were also collected every week during July-September 2021 from a natural population located on the banks of Grand River in Waterloo, Ontario (Claude Dubrick trail, 43°30’11.40"N, 80°29’37.48"W). This is not a conservation or protected area and *C*.*gronovii* var. *gronovii* is the most common dodder in Canada. Herbarium vouchers for all the living *Cuscuta* species used are kept at Wilfrid Laurier University herbarium.

### Inflorescence development

Inflorescence development was observed on the living plants until they finished their life cycle. Fresh samples were examined and imaged under a Nikon SMZ1500 stereo microscope, and then using scanning electron microscopy. Leaf scales or bracts at the base of cymes were removed to reveal incipient stages of axes or flower development within the inflorescences. Dissected samples were dehydrated through series of ethanol (30%, 50%, 75%, 85%, 95%, and 100%) for at least 30 minutes each, then critically point dried with Tousimis Autosamdri-931. Samples were sputter-coated with 30 nm of gold using an Emitech K550 (Emitech, Ltd. Ashfort, UK). Imaging was conducted with a Hitachi SU-510 variable pressure scanning electron microscope (SEM) at three kV. Measurements were conducted using Quartz PCI version 5.1 (Quartz Imaging Corp.).

### Character evolution and statistical analyses

The terminology used to describe the inflorescence in *Cuscuta* was compiled from literature [2, 3, 33–48] and the different types were reassessed based on the examined material to generate qualitative character-states. Cymose inflorescence type were reviewed from the definitions and interpretations of [21–24, 26, 29, 67–69].

Two continuous inflorescence characters were measured from herbarium specimens—the total axes length (not including the pedicel length) and the longest pedicel length. In addition, the flower tube length and flower diameter were measured. Pollen-ovule ratios were used as a proxy of breeding systems based on Cruden’s mating system categories [70] and pollen-ovule data were obtained from [15]. New pollen-ovule counts were obtained for ten additional taxa (nine species and one variety) using the same methodology as [15]: *Cuscuta bonafortunae*, *C*. *californica* var *papillosa*, *C*. *colombiana*, *C*. *globulosa*, *C*. *liliputana*, *C*. *membranacea*, *C*. *polygonorum*, *C*. *psorothamnensis*, *C*. *timida*, and *C*. *tolteca* (herbarium specimens used were indicated with an asterisk in S1 Appendix). The fruit dehiscence modes data were taken from [19]: dehiscent (DE; with an abscission zone at the base of capsules), indehiscent (IN; abscission zone is absent), irregularly dehiscent type A (IrA; no abscission zone present but the pericarp is thin at the base and tears irregularly) and irregularly dehiscent type B (IrB; are structurally IN, but they break irregularly because of the forces created within dense infructescences).

The relationships between the inflorescence traits, flower variables and pollen-ovule ratios were analyzed by calculating Spearman’s rank coefficient of correlation in R (version 4.0.3, [71]). A Principal Component Analysis (PCA) was performed on the inflorescence variables, the flower diameter and the corolla length data, based on their correlation matrix. The relationship between the PCA-rescored variables and the pollen-ovule ratios and the fruit widths were then analyzed using Spearman’s rank coefficient. The median total axes, pedicel lengths, fruit widths and fruit lengths between groups differing in mode of dehiscence were compared using Kruskal-Wallis rank sum tests. The location of specific difference between group medians was determined using the *kruskalmc* function in the *pgirmess* package [72].

The total axes and pedicel lengths, as well as the types of inflorescences observed (see Results) were mapped onto a strict consensus genus phylogeny based on *rbcL* and nrLSU sequences [73]. Analysis of character polarity in *Cuscuta* using formal outgroup analysis is hindered by the unresolved position of *Cuscuta* in Convolvulaceae [74]. Thus, to reconstruct ancestral character states in *Cuscuta*, the distribution of character states was analysed only in-group, similar to previous character evolution studies [14, 16–20]. Scenarios of character evolution were analysed using the parsimony and likelihood reconstruction methods implemented in Mesquite 3.2 [75]. Markov k-state 1 parameter model (MK1) model of evolution was used; in the parsimony reconstruction, character-state changes were treated as unordered.

## Results

### Inflorescence development and major architectural types in *Cuscuta*

Quantitative data obtained/used in this study were summarized in S2 Appendix. SEM imaging data did not reveal any additional details compared to stereomicroscopy, and was not included in the results.

*Cuscuta* has two functional types of shoot systems: exploratory, which forage in the plant community, and haustorial, which twine in a dextrorse direction around the stems of the hosts and produce haustoria. Vegetative branching occurs especially in the exploratory shoots and is sympodial. One to four (six) shoots can emerge successively from axillary buds at the base of the cauline scale-like leaves, which have a 2/5 phyllotactic alternate arrangement. In subgenera *Monogynella* and *Cuscuta*, “floral unit meristems” sensu [29] develop extensively on the exploratory stems, while in subgenera *Pachystigma* and *Grammica*, most of the cymes are concentrated on the haustorial stems.

Inflorescences are axillary; the primary axes originate from buds located underneath the reduced, cauline scale-like leaves, while the subsequent, higher order axes, arise from buds found underneath bracts. The inflorescence fundamental unit in all *Cuscuta* species is a monochasial cincinus or scorpioid cyme, and different architectural patterns emerge depending on whether the cymes are simple or compound, and contingent on the number of orders and the length of different axes. Inflorescence axes and pedicels attain maximum length at anthesis and remain size-invariant at fructification. Thus, in *Cuscuta*, infructescences are identical to inflorescences.

Two main rules that allow decoding of the inflorescence patterns are: 1) scale-like leaves or bracts always indicate a branching point and the origin of a cyme. This is made evident by their removal, which reveals underneath the flower buds at different stages of development. 2) The oldest flowers are the farthermost from the leaf scales or bracts. In the case of the compound cymes, the oldest cymes are the farthermost from the leaf-scale. Three major types of developmental patterns are evident in *Cuscuta*, and characterize subgenera.

**Fig 1:**
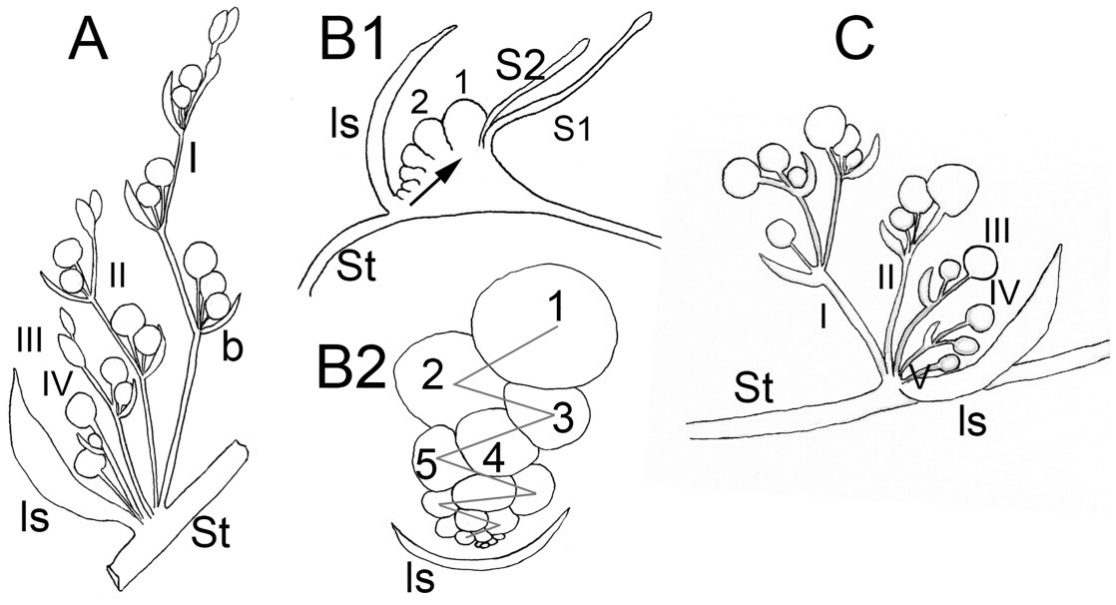
Diagrams of major types of inflorescences in *Cuscuta*. A. “*Monogynella*” type is a compound monochasial scorpioid cyme in which the main axes (e.g., I and II) superficially resemble thyrses. B. “*Cuscuta*” type is a simple scorpioid cyme, in this case with sessile flowers. B1. Simple cyme seen laterally (arrow indicates the direction of flower development). B2. Cyme seen from the top. C. “*Grammica*” type is also a compound monochasial scorpioid cyme, but with shorter axes. b = bract; ls = leaf scale; I, II, III indicate the order of axes.

**Fig 2:**
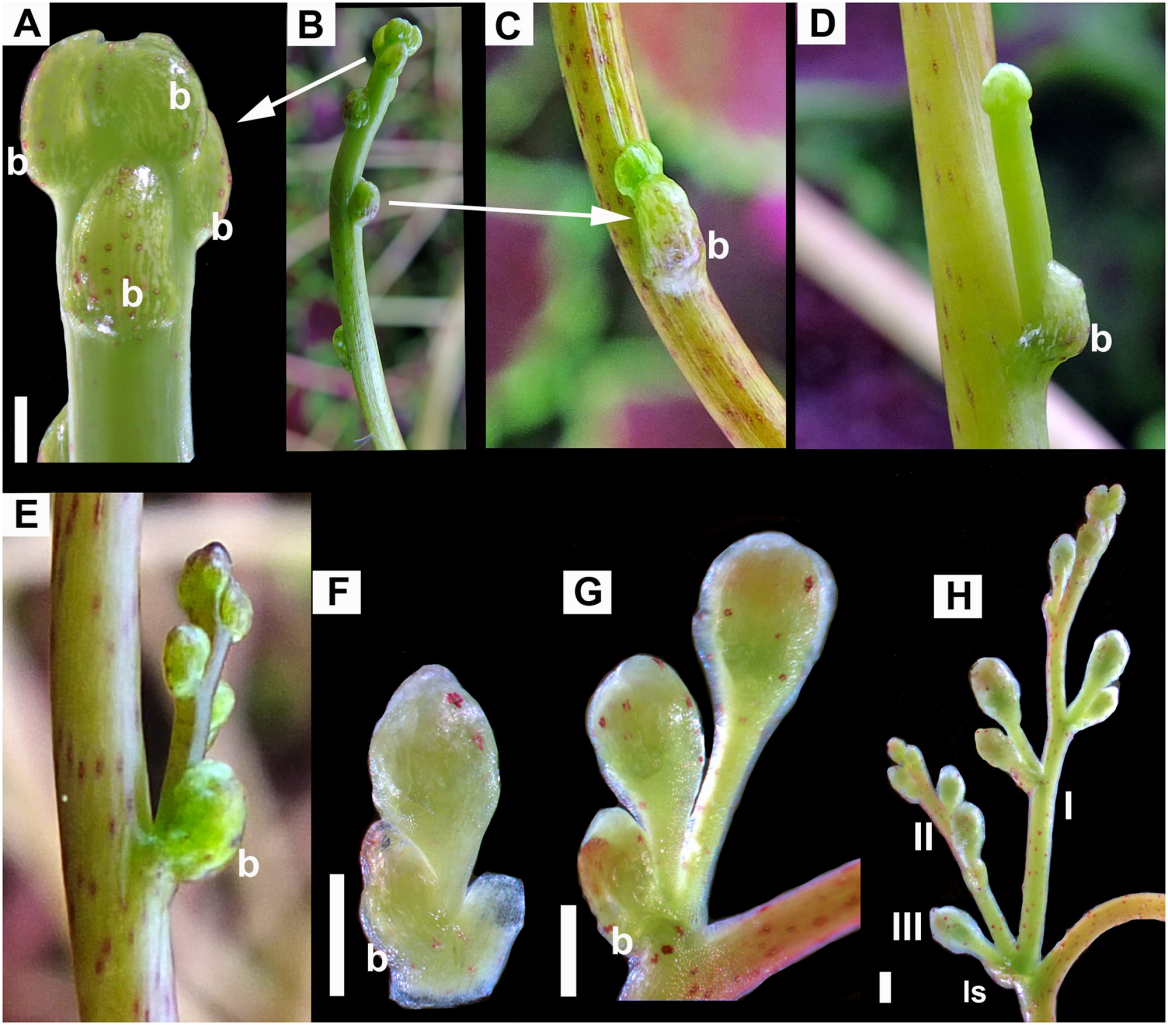
Development of “*Monogynella*” type of inflorescence. A, B. The first inflorescence axis. A. Apical bud that grows vegetatively and generates lateral monochasial cymose axes. B. General view of first inflorescence axis. C–E. Progressive stages in the development of secondary axes. F, G. Simple scorpiod cymes developing on the secondary axes. H. More advanced stage in the development of the overall compound scorpioid cyme. I, II, III indicate axes of different orders. b = bracts; ls = leaf scale. Scale bars = 1 mm.

“*Monogynella*” type (Figs 1A, 2, 5A, 5B). Inflorescences in species of subg. *Monogynella* are compound monochasial scorpioid cymes. Characteristic to this type is the prolonged growth of the longest primary axes, which superficially imparts them the appearance of thyrses (racemes or spikes with cymes).

**Fig 3:**
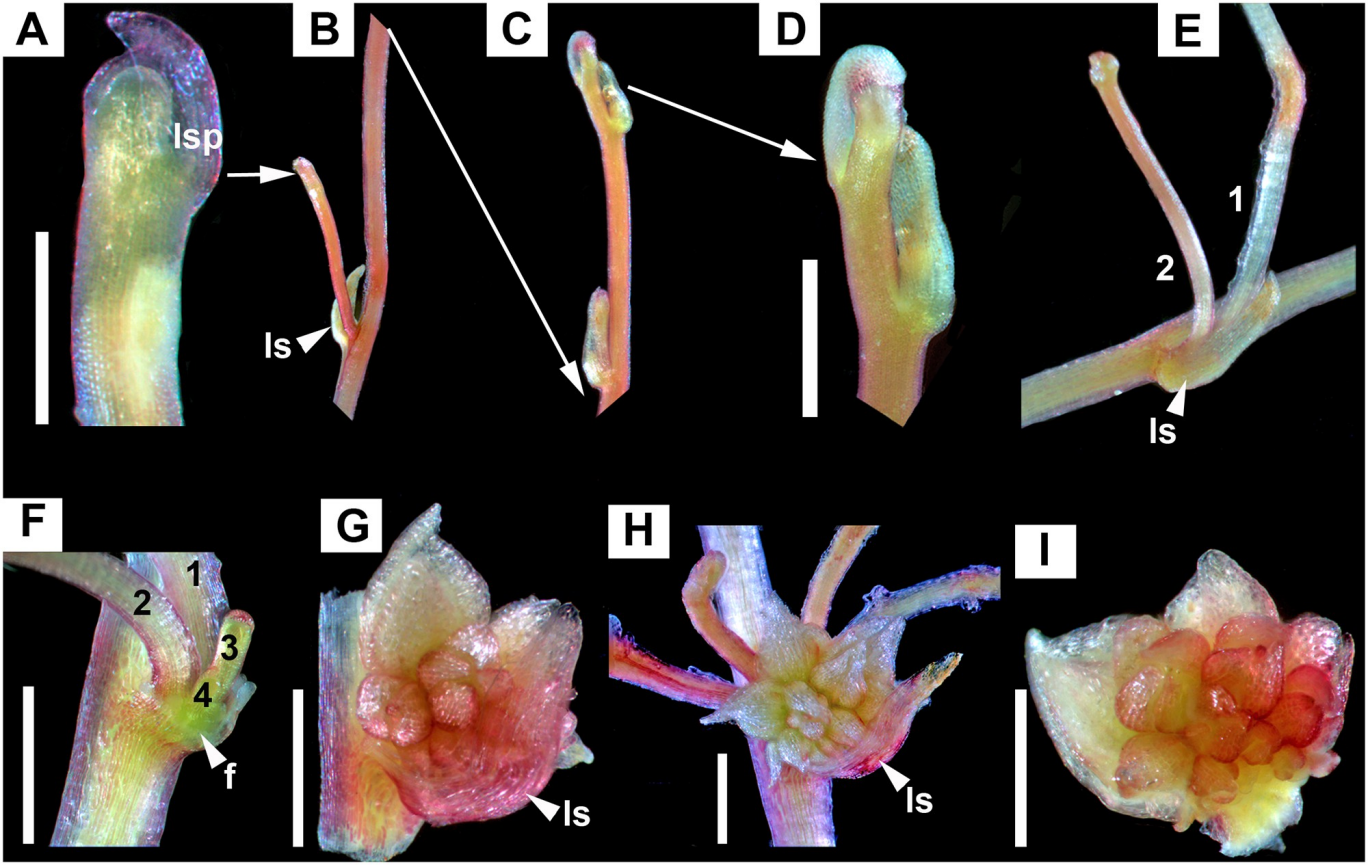
Development of shoot system and “*Cuscuta*” type of inflorescence. A–E. Sympodial development of exploratory shoot system. A–D. Axillary exploratory shoot. A. Apex of lateral exploratory shoot. B. Fragment of (primary) exploratory shoot showing axillary (secondary) developing exploratory shoot. C, D. Distal part of exploratory shoot from B. E. First two orders of exploratory shoots. F–I. Development of inflorescence. F. Incipient stage of flower primordia emerging at the base of series of axillary exploratory shoots (1, 2, 3, 4). G–I. Sessile flowers developing in zig-zag to form a simple scorpioid monochasial cyme (leaf scale removed from I). ls = leaf scale; lsp = leaf scale primordia; f = flower. Scale bars = 1 mm.

“*Cuscuta*” type (Figs 1B, 3, 5C–5E). The species of subg. *Cuscuta* examined in this study have sessile flowers developing on a small, common receptacle, under a single leaf scale. The dense, spherical inflorescences superficially resemble a capitulum (but which is a racemose type of inflorescence). Flower formation follows a zig-zag pattern, with the youngest flowers emerging closest to the leaf scale and the oldest located farthest away, the inflorescence being a simple scorpioid cyme.

**Fig 4:**
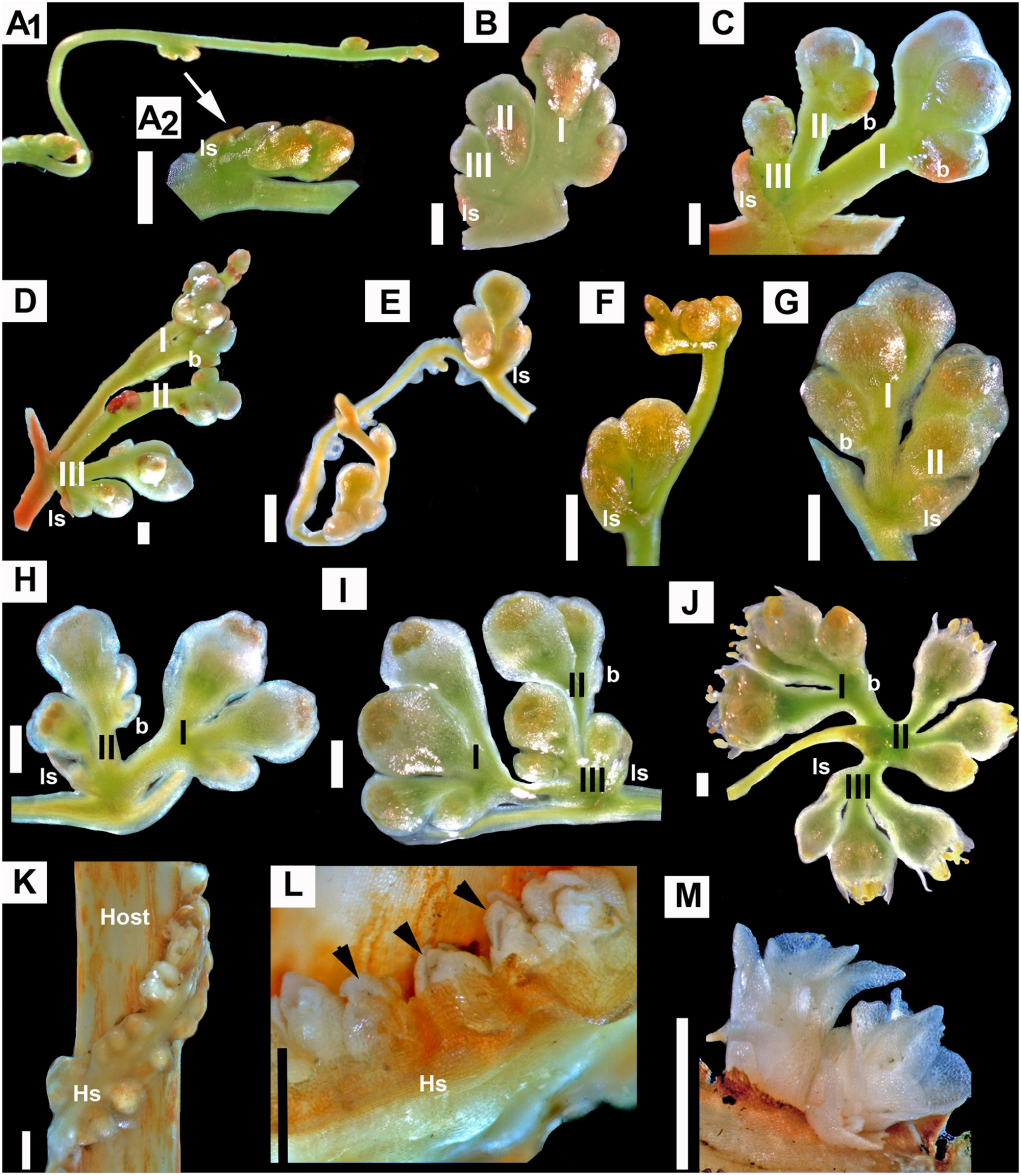
Development of “*Grammica*” type of inflorescence. A–D. *Cuscuta gronovii*. A1. Shoot. A2. Incipient stage of axillary inflorescence. B–D. Progressive stages in the development of compound monochasial scorpioid cyme resembling a panicle. E–J. *Cuscuta campestris*. Different stages of “glomeruliform-subglomeruliform” inflorescence development. K–M. “Rope” inflorescence of *Cuscuta glomerata*. K. General view of early stage. L. Flower primordia (indicated with arrows) emerging from haustorial stems. M. Developing flowers. ls = leaf scale; b = bract; Hs = haustorial stem; I, II, II, orders of axes; scale bars = 1 mm.

**Fig 5:**
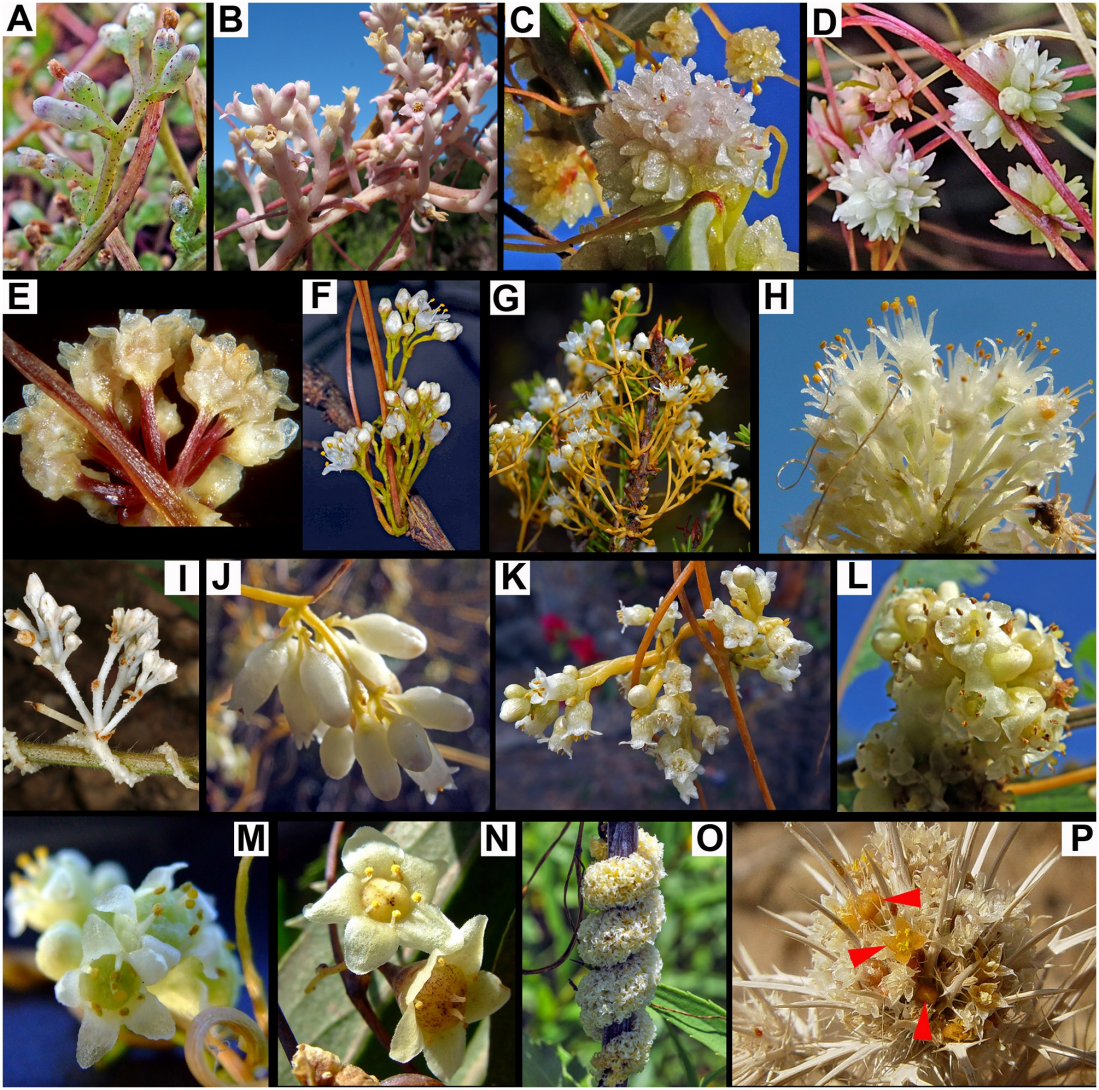
Variation of inflorescence architecture in *Cuscuta*. A, B. Subgenus *Monogynella*. A. *Cuscuta lupuliformis*. B. *C*. *lehmanniana* (photo by Vladimir Kolbintsev). C–E. Subgenus *Cuscuta*. C. *C*. *planiflora*. D. *C*. *epithymum*. E. *C*. *babylonica* (photo by Miguel García). F, G. Subgenus *Pachystigma*, “umbelliform-corymbiform” inflorescences. F. *C*. *africana* (photo by Miguel García). G. *C*. *angulata* (photo by Miguel García). H–P. Subgenus *Grammica*. H, I. “Umbelliform-corymbiform” inflorescences. H. *C*. *sidarum*. I. *C*. *erosa* (photo by Jillian Cowles). J–K. “Racemiform- paniculiform” inflorescences. J. *C*. *corymbosa* var. *grandiflora*. K. *C*. *tinctoria* var. *floribunda*. L, M. “Glomeruliform -subglomeruliform” inflorescences. L. *C*. *volcanica*. M. *C*. *obtusiflora* var. *glandulosa*. N. Flowers solitary or in fascicles, *C*. *grandiflora*. O. “Rope”, *C*. *glomerata* (photo by Richard Lutz). P. Mimetism of *C*. *howelliana* inflorescence developed inside the inflorescence of *Eryngium castrense* (Photo by Carol Witham).

“*Grammica*”-type (Figs 1C, 4, 5F–5P). The inflorescence of all the species of subg. *Pachystigma* and most species of subg. *Grammica* is a compound monochasial scorpioid cyme with up to five orders of axes. In contrast to the “*Monogynella*” type, the primary axes have a limited growth, and thus, the semblance to a thyrse is not apparent.

The variations encountered in subg. *Grammica* refer to the number of orders, the number of axes per order, and their length relative to the length of the pedicels. *Grammica* type is the most diverse within genus *Cuscuta* (Fig 5). Based on their superficial resemblance to racemose inflorescences, following the literature, the compound cymes of subgenera *Pachystigma* and *Grammica* were further informally categorized as “umbelliform-corymbiform” (Fig 5F–5I) and “racemiform- paniculiform” (Fig 5J and 5K) when flowers were long-pedicelled. Compound cymes with sessile or very short pedicellate flowers were scored as “glomeruliform -subglomeruliform” (Fig 5L and 5M).

Exceptions of solitary flowers or simple scorpioid cymes with just a few flowers (“fascicles”) evolved only in a few species from several clades of subg. *Grammica* (Fig 5N; see next section). Another exception within subg. *Grammica* is the “rope”, which is a unique flower-aggregation architecture that was observed in *C*. *glomerata* (sect. *Oxycarpae*). Flower buds emerge endogenously directly from the haustorial shoots, irrespectively to the reduced leaves (Fig 4K–4M). No axes or pedicels develop and, as a result, the parasite stem appears as a dense “rope” of flowers twining around the stem of the host (Fig 5O).

### Inflorescence character evolution

**Fig 6:**
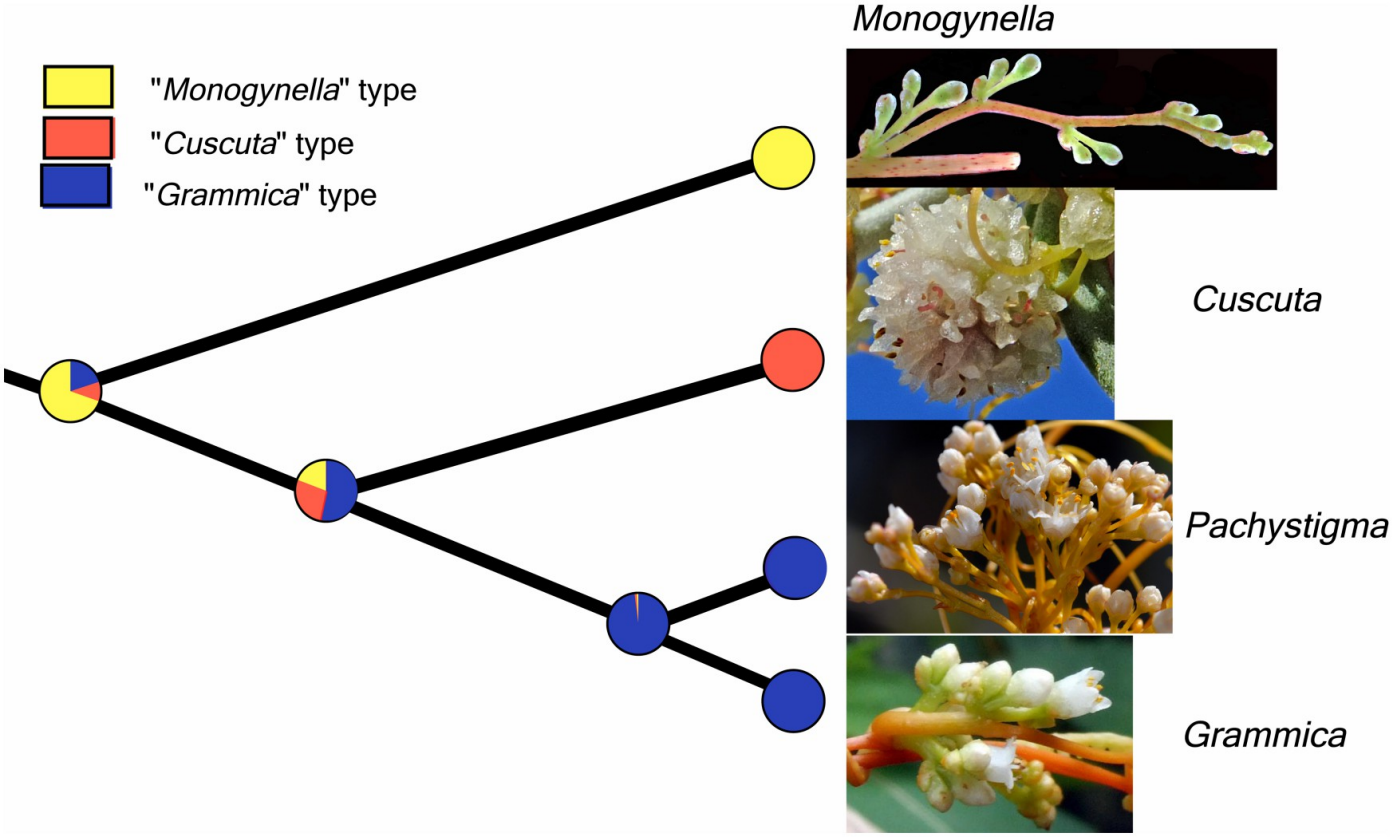
Summary of subgeneric character evolution hypotheses for the three main types of inflorescences using likelihood. “*Monogynella*” type was suggested to be ancestral (proportional likelihood = 0.6962), followed by “*Cuscuta*” and “*Grammica*” types.

In a first reconstruction of ancestral character states only the three major types of inflorescences were scored. Likelihood reconstruction suggested that “*Monogynella*” type is ancestral (proportional likelihood = 0.6962; Fig 6). At the second node, “*Grammica*” type had a higher likelihood (proportional likelihood = 0.5328; Fig 6) compared to the other two types of inflorescences. In the parsimony reconstruction, all three major types were indicated as most parsimonious (MPR) at the first and second node; with *Grammica* type being MPR only at the third node (results not shown).

**Fig 7:**
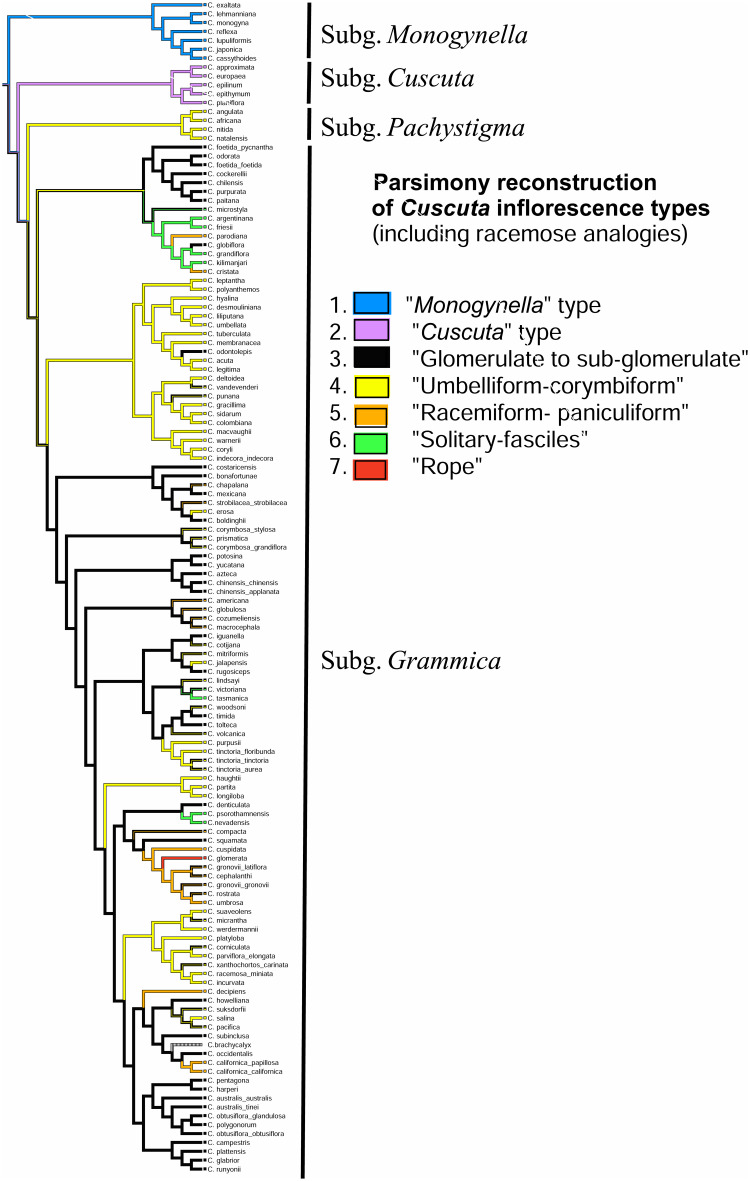
Parsimony reconstruction of all the types of inflorescences, including the racemose analogies in subg. *Pachystigma* and *Grammica*, revealed extensive convergent evolution in the latter subgenus.

In a second character evolution analysis, we also added as character states “rope”, “solitary or fascicles” as well as the morphologies resembling racemose types for the subg. *Pachystigma* and *Grammica* (Fig 5). Some species of subg. *Grammica* possess inflorescences that correspond to multiple character states (polymorphism), and only the parsimony reconstruction could be used. The four species of subg. *Pachystigma* examined are all “umbelliform-corymbiform” (Fig 7). While at the first nodes of subg. *Grammica*, both “umbelliform-corymbiform” and “glomeruliform-subglomeruliform” are indicated as MPR, at the deeper nodes, only the latter dominate (Fig 7). Overall, each of 15 clades of subg. *Grammica* is characterized by one or two major “types” accompanied sometimes by the recurrent evolution of a third “type”. The most common type of inflorescence in subgenus *Grammica* is glomeruliform-subglomeruliform. “Solidary-fascicle” evolved in three major clades of subg. *Grammica* (sections *Subulatae*, *Lobostigmae* and *Denticulatae*; Fig 7). A convergent trend of shortening of the total axes was noticeable at the scale of the entire genus (S1 Fig), but this trend was not observed for the length of the pedicels (S2 Fig).

### Correlations between inflorescence, flower, pollen-ovule ratios and fruit variables

Weak (< 0.3) but statistically significant positive correlations were observed between the flower diameter and pedicel length; and pollen-ovule ratios and total axes length (Table 1). Moderate (0.3–0.6) significant correlations were observed between total axes length and pedicel length; pollen-ovule ratios and flower tube length; pollen-ovule ratios and flower diameter; fruit width and flower tube length; and fruit width and flower diameter (Table 1). All the other correlations were not statistically significant (Table 1).

**Table 1:** Spearman’s correlation between all the inflorescence variables examined as well as pollen-ovule ratios, and fruit width. Asterisk indicates significant p-values (p-value < 0.05).

	**Pedicel Length**	**Total axes length**	**Corolla tube length**	**Corolla diameter**	**Pollen-ovule ratio**	**Fruit width**
**Pedicel Length**	1.00	0.50*	0.032	0.21*	0.10	-0.08
**Total axes length**		1.00	0.17	0.15	0.19*	0.11
**Corolla tube length**			1.00	0.42	0.51*	0.36*
**Corolla diameter**				1.00	0.48*	0.36*
**Pollen-ovule ratio**					1.00	0.10
**Fruit width**						1.00

### Principal component analysis of inflorescence traits and correlations with pollen-ovule ratios, and fruit width

**Fig 8:**
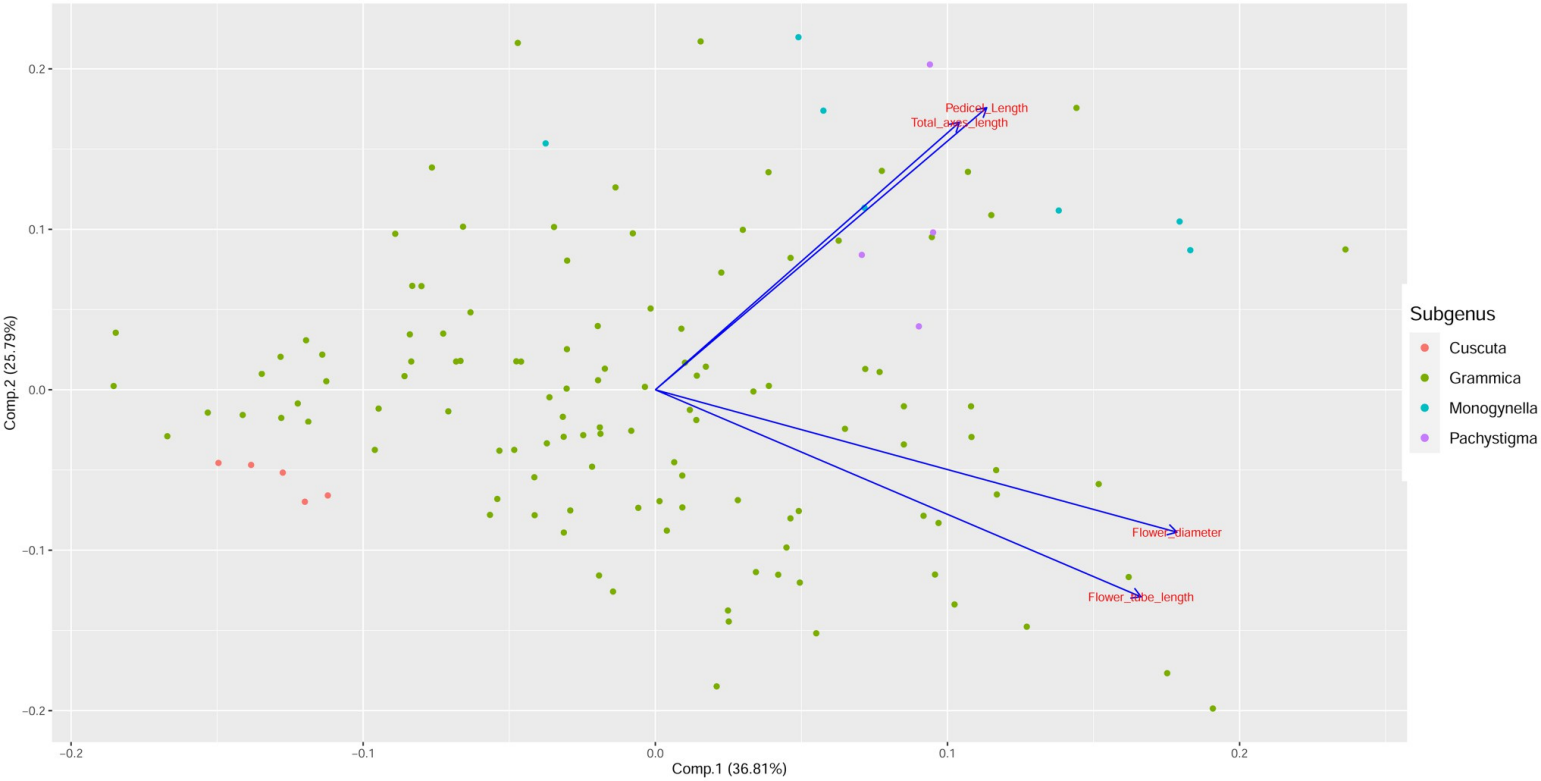
Principal component analysis (PCA) biplot of component 1 and component 2 using the inflorescence variables. Colour groups show the different subgenera in *Cuscuta*.

Principal Component 1 accounted for 36.81% of the variance and component 2 accounted for 25.79% of the variance (Table 2). Cumulatively, both components 1 and 2 accounted for 62.6% of the variance. The biplot showed that all the variables had positive loadings on the first component, with corolla diameter and corolla tube length having larger positive loadings than the pedicel length and total length of axes (Fig 8). Therefore, component 1 can be regarded as a general flower size-related variable, with taxa that have large positive PC1 scores exhibiting on average larger corolla diameter, longer corolla tubes, total axes and pedicels, while taxa with large negative principal component scores showing on average shorter corolla tubes and smaller corolla diameters and shorter total axes/pedicels.

**Table 2:** Loadings for all the variables with the principal components.

	Component 1	Component 2	Component 3	Component 4
**Pedicel length**	0.393	0.609	0.595	0.347
**Total axes length**	0.361	0.578	-0.693	-0.237
**Flower tube length**	0.576	-0.447	-0.285	0.622
**Corolla diameter**	0.619	-0.308	0.292	-0.661

The second principal component had positive loadings for pedicel length and total axes length, and negative loadings for corolla diameter and corolla tube length (Table 2). Therefore, component 2 can be seen as an overall axes length-related variable; taxa that have large positive principal component 2 scores, on average, have longer pedicels and longer total axes, but smaller corolla diameter and shorter corolla tubes, while taxa with large negative principal component 2 scores tend to have shorter pedicels, and shorter total axes, but longer corolla tubes and larger corolla diameter.

**Fig 9:**
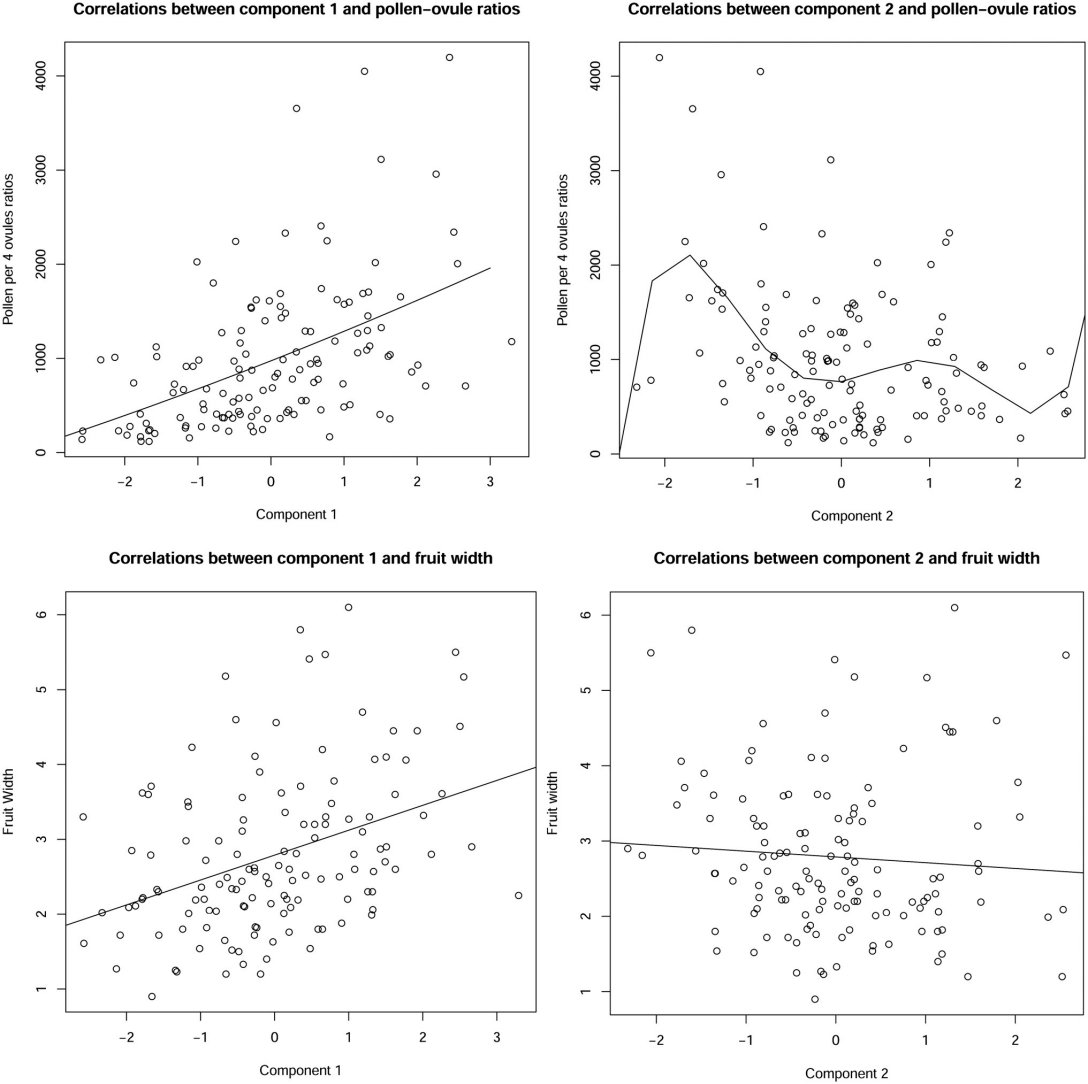
Correlation plots between components and reproductive traits. A. Between component 1 and pollen-ovule ratios. Equation of the line of best fit: PO = 7.655 × (Comp 1)^2^ + 305.662 × (Comp 1) + 974.417. B. Between component 2 and pollen-ovule ratios. Equation of the line of best fit: PO = 54.93 × (Comp 2)^5^–80.28 × (Comp 2)^4^–358.95 × (Comp 2)^3^ + 448.34 × (Comp 2)^2^ + 162.83 × (Comp 2) + 765.47. C. Between component 1 and fruit width. D. Equation of the line of best fit, Fruit width B = 0.3327 × (Comp 1) + 2.7887. Equation of the line of best fit, fruit width B = −0.07643 × (Comp 2) + 2.78871.

Using principal components 1 and 2, correlations between the principal component scores and pollen-ovule ratios, and fruit width were examined using Spearman’s correlation. Statistically significant positive correlations were observed between both component 1 scores and pollen-ovule ratios, and with fruit width (Fig 9A and 9C). Pollen-ovule ratios had the largest positive correlation with a rho value of 0.533 (Table 3). This indicates a pattern according to which larger flowers with longer pedicels and total axes also have large pollen-ovule ratio values, and fruit widths.

**Table 3:** Spearman’s correlation between principal component 1 and principal component 2 with pollen-ovule ratios, and fruit width. Asterisk indicates significant p-values (p-value < 0.05).

	Component 1		Component 2	
	rho	p-value	rho	p-value
**Pollen-ovule ratios**	0.533	4.764e-11*	-0.259	0.002712*
**Fruit width**	0.388	4.492e-06*	-0.143	0.1022

Negative correlations were observed between component 2 scores and both pollen-ovule ratios, and fruit width (Fig 9B and 9D). The correlations for pollen-ovule ratios and component 2 were significant, but the correlation between fruit width and component 2 was not statistically significant (Table 3). Therefore, taxa with small flowers, long pedicles and total axes lengths tend to have smaller pollen-ovule ratios, while the size of their fruits is largely independent.

### Relationships between fruit variables and between inflorescences and fruit

**Fig 10:**
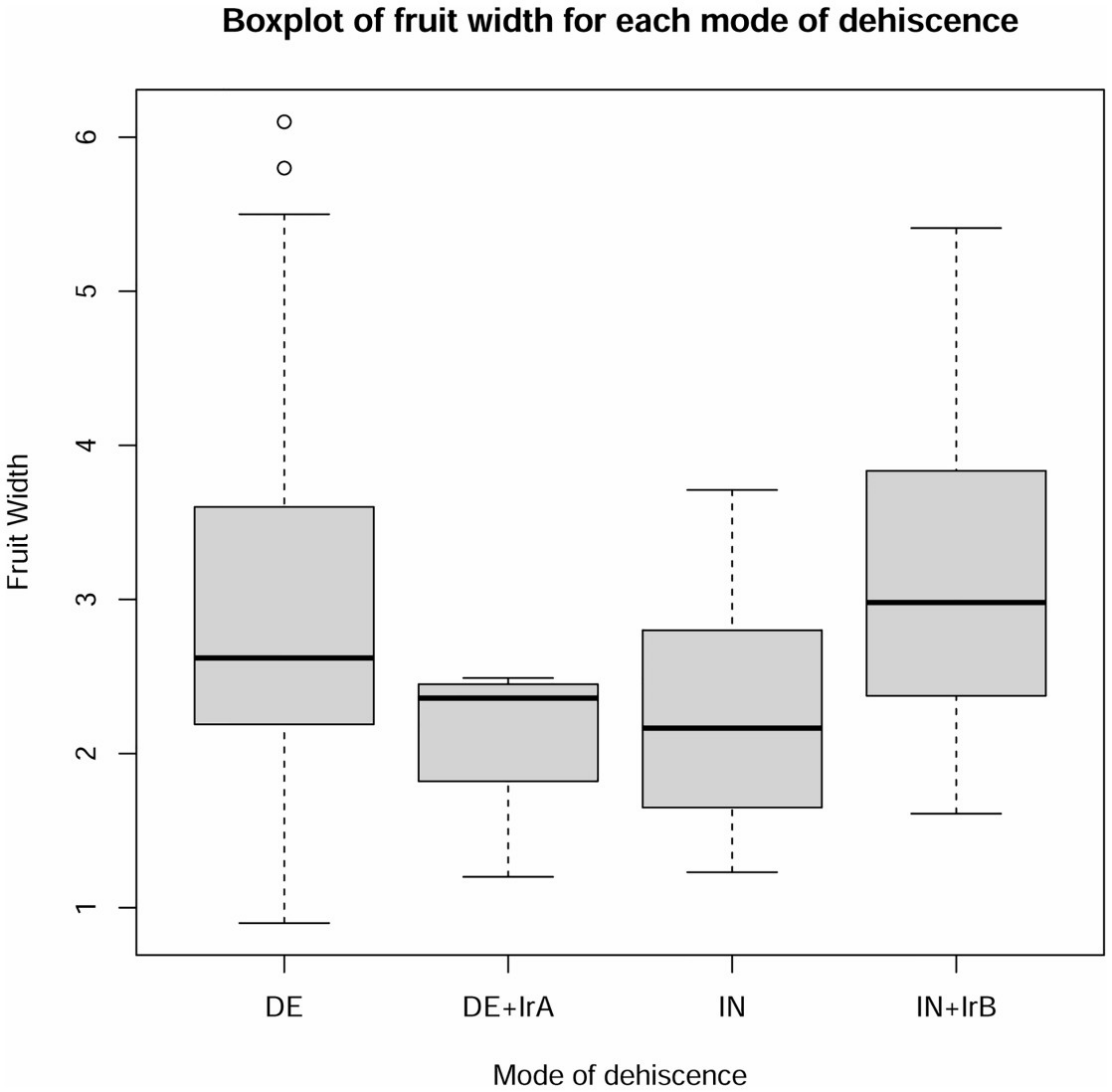
Boxplot of fruit width for DE, DE+IrA, IN, and IN+IrB modes of dehiscence (3 groups were removed as they had 3 taxa or less). Boxes show the middle 50% of component 1 values for the modes of dehiscence, horizontal lines in the boxes show the median, circles are outliers, and the whiskers show the minimum and maximum values. Significant differences were noted for at least two of the groups of dehiscence examined. Pairwise comparisons using Wilcoxon rank sum test revealed significant differences between IN and DE (p-value = 0.033), and IN+IrB and IN (p-value = 0.033).

A Kruskal-Wallis Rank Sum test was performed to compare the effect of the mode of dehiscence on median fruit width and length. Fruit dehiscence character states dehiscent (DE), dehiscent and irregular dehiscent type A (DE + IrA), indehiscent (IN), and indehiscent and irregularly dehiscent type B (IN + IrB) were included as categorical variables. Overall, we rejected the null hypothesis that the median fruit width was the same across all dehiscence groups (Kruskal-Wallis χ^2^ = 11.69, p-value = 0.009). Pairwise comparisons indicated that the only significant differences in median fruit widths were located between IN and DE groups, and between IN + IrB and IN groups (Fig 10). Overall, no statistically significant differences were found between modes of dehiscence and median fruit length scores (χ^2^ = 6.689, p-value = 0.082), nor between median pedicel length and the modes of dehiscence groups (χ^2^ = 5.603, p-value = 0.133).

## Discussion

### *Cuscuta* inflorescence; taxonomic and systematic significance

This study confirmed the early insightful observations of Mirande [30] in that the fundamental unit of inflorescence in *Cuscuta* is a scorpioid or cincinnus monochasial cyme. Mirande [30] deserves more recognition, not just for the inflorescence architecture, but also for revealing the first details of stem anatomy and stomatiferous protuberance in *Cuscuta* [18]. From an ontogenetic point of view, the inflorescences of *Cuscuta* originate from “floral unit meristems” as defined by [29]. The inflorescence in subg. *Monogynella* is not a thyrse as previously thought [4], but a compound monochasial scorpioid cyme, which could be revealed only by examining the development of the inflorescence in this subgenus. All the species of subg. *Cuscuta* in this study have dense, globose, simple scorpioid cymes, with sessile or short-pedicellate flowers superficially resembling more (racemose) capitules than glomerules. However, three species not studied here, *C*. *babylonica*, *C*. *pulchella* and *C*. *rausii* are known to have pedicellate flowers ([32, 34]; Fig 5E), which imparts them a loose glomeruliform or umbelliform aspect.

Although it was possible to define three major types of inflorescences that characterize subgenera *Monogynella*, *Cuscuta* and *Grammica* (together with *Pachystigma*), the quantitative inflorescence traits (total length of axes and pedicels) varied throughout the genus, and sometimes even within the same species. Similarly, the racemose type analogies were convergent within different clades of subg. *Grammica* and sometimes polymorphic within the same species. The extent of convergent evolution observed for the inflorescences is not surprising because the majority of morphological traits in *Cuscuta* exhibit convergent evolution and polymorphism. Studies of other morphological characters in *Cuscuta—*perianth features [15]; shape, size and reduction of infrastaminal scales [16], pollen morphology [17], gynoecium characteristics [14, 73], multicellular protuberances with stomata [18], and modes of fruit dehiscence [19]—uncovered relatively few traits with strong phylogenetic signal (e.g., the number of styles and shape of stigmas, the seed coat morphology and anatomy).

Despite their convergent evolution, the racemose analogies are useful to separate species within the same major clade of subg. *Grammica*, which gives them taxonomic value as proven by their use in numerous identification keys and species descriptions publications e.g., [2, 3, 33–48]. Although they are technically incorrect, racemose analogies are easier to observe and describe than the different components of monochasial cymes, and we anticipate that they will remain useful and more practical for the species-level taxonomy of the genus.

### Inflorescence architecture in solanaceae and convolvulaceae

A recent study on the evolution and development of inflorescences in Solanaceae, the sister family of Convolvulaceae in Solanales (e.g., [76, 77], showed that both scorpioid and dichasial cymes are present in the family, and the latter are likely derived from the former [78]. Within Convolvulaceae, no comparative or character evolution inflorescence study has been conducted either at the level of the entire family or in different genera. However, based on the descriptions and illustrations available, the inflorescences are cymose, terminal or axillary; simple or compound, monochasial, dichasial, sometimes with both of the latter patterns encountered within the same compound inflorescence or on the same plant, and only rarely flowers are solitary (e.g., [79–82]. Inflorescences were used for the separation of major intrafamilial groups, genera [82, 83], or more commonly species (e.g., [81, 84]). Similar to *Cuscuta* (see above), racemose analogies (e.g., “panicle”, “raceme”, “umbelliform”) are more often used in species descriptions than the cymose terminology (e.g., [85, 86]). Cincinnus was employed for the description of the inflorescence unit of Convolvulaceae [87] and scorpioid cymes were mentioned in *Maripa* and *Dicranostyles* [81], *Jacquemontia* [88] and *Erycibe* [89]. The overall similarity and homology of *Cuscuta* inflorescence to Convolvulaceae supports the inclusion of the genus in this family, in contrast to attempts to recognize *Cuscuta* as a distinct family based on its parasitic nature (e.g., [90, 91]).

### Relationship between inflorescence/flower traits and pollen-ovule ratios

With the exception of the pedicel length, which had a low correlation with the flower diameter, no other significant correlations were observed between inflorescence traits, pollen-ovule ratios and flower size (Table 1). Interestingly, the total axes and pedicel lengths were also not correlated, suggesting an independent variation. In the PCA, positive significant correlations for component 1 and pollen-ovule ratios, suggested that species possessing inflorescences with longer pedicels, longer total axes, longer corolla tubes and wider flowers should on average have larger pollen-ovule ratios. This relationship, nevertheless, was not universal in *Cuscuta*. Pedicel length and total axes length had positive loadings in both components of the PCA, but the flower size variables had contrasting, positive or negative loadings in the first and second components, respectively. Some species with very high pollen-ovule ratios did not have long pedicels or axes, nor did species with the shortest pedicels or total axes length had the lowest pollen-ovule ratios. For example, *C*. *volcanica* (sect. *Lobostigmae*, subg. *Grammica*) (Fig 5L) had the highest pollen-ovule ratio in the genus (4197, [15]), which strongly implied obligate xenogamy. This species has glomerulate inflorescences, with short axes and pedicels [48]. Most of the species of sect. *Lobostigmae* (Subg. *Grammica*) have high pollen-ovule ratios [15] and compact, often glomerulate inflorescences [48]. In contrast, another species with a similar glomerulate inflorescence architecture, *C*. *obtusiflora* var. *obtusiflora* (sect. *Cleistogrammica*, subg. *Grammica*; [4, 39]) (Fig 5M) had one of the lowest pollen-ovule ratios in the genus (228; [15]), and has been suggested to be functionally cleistogamous with the ovules self-fertilized before the flowers open [92]. The morphological difference between the mentioned species lies in the size of the flowers, which are small in *C*. *obtusiflora* (1.8–2.5 mm; [39]) and large in *C*. *volcanica* (7–9 mm long; [48]). In general, flower size variables are a better predictor of breeding systems in *Cuscuta* than inflorescence traits.

Pollinators may act as selection agents for the diversification of reproductive morphological traits in angiosperms, including their inflorescences (e.g., [93–96]). *Cuscuta* species often synchronize their flowering with the hosts [10, 97, 98] and they share the same pollinator niches with them. For example, *C*. *epithymum* (subg. *Cuscuta*) populations growing in heathlands from Belgium share the same generalized hymenopterans and syrphid fly pollinators [99] with their co-flowering host, *Calluna vulgaris* (Ericaceae) [100]. Similarly, *C*. *epithymum* from Sierra Nevada mountains in Spain takes advantage of the pollinators of its host, *Hormathophylla spinosa* (Brassicaceae) [101]. An even more extreme case is that of *C howelliana* (subgenus *Grammica*), which parasitizes *Eryngium* species from alpine vernal pools in California [41]. The scorpioid cymes of *C*. *howelliana* (Subg. *Grammica*, sect. *Californicae*; [41]) develop inside the inflorescences of its host, and the small flowers of the parasite mimic those of the host and synchronize their anthesis with them, ensuring the use of the same pollinator system.

Host ranges in *Cuscuta* vary from narrowly specialized to different degrees of “generalist” strategies (e.g., [11, 37, 102]). Especially in the later case, which comprises the majority of *Cuscuta* species, the host range is dynamic as different dodders may shrink or expand their geographical distribution ranges and ecological niches. The constant interaction with a diversity of hosts may prevent the morphological selection to particular pollination host niches, maintaining the inflorescence architecture diverse throughout the genus and sometimes within the same species. It is possible that the most widespread and common *Cuscuta* species worldwide, which can inhabit different habitats, may produce different inflorescence architectures corresponding to the host plant communities of those habitats. At the same time, this may explain the presence of contrasting pollen-ovule ratios in species with the same inflorescence architecture. Pollen-ovule ratios were also not correlated with the inflorescence architecture in other plants, e.g., *Philodendron* and *Anthurium* [103] and *Tradescantia* [104].

### Relationship between inflorescence and the fruit traits

This study supported previous anecdotal observations made in subg. *Grammica* according to which the inflorescences of species with indehiscent fruits are “denser”, whereas species with regularly circumscissile capsules possess more commonly lax inflorescences [4, 19, 39]. The “density” effect is generated by the varying lengths of the total infructescence axes, but not of the pedicels for which no significant differences were found between the modes of dehiscence. Since glomerulate inflorescences have shorter axes, the fruits that develop in such compact infructescences will be more densely packed. The pressures created result in the irregular dehiscence of some of the indehiscent capsules, which will affect both the dispersal and germination of seeds. Indehiscent fruits of *Cuscuta* disperse as units (diaspores) that include up to four seeds, and are capable of long-distance dispersal by water [19]. Also, seeds in indehiscent fruits exhibit a gradual and extended germination pattern. In contrast, dehiscent fruits release all their seeds, which usually remain in the vicinity of the plants, where they will mass-germinate during one or two seasonal peaks [19]. Species with dense infructescences in which some of the fruits open irregularly can thus benefit from the advantages conferred by both dehiscence modes. Because germination behaviour is different in dehiscent and indehiscent fruits, this situation resembles the heterodiaspory condition, in which two different morphological types of diaspores differing also in ecological function are produced by the same plant (reviewed by [105]).

Infructescence architecture has also been associated with dispersal in other plant species. In *Lepidium campestre*, the height of seed release (which was a function of plant height and infructescence length) was noted to affect the skewness and kurtosis of seed number distribution [106]. As well, in some *Phyteuma* species, the seed output rate was influenced by the infructescence architecture (Maier et al. 1999). The latter study also showed that characteristics of fruits, infructescences, and seed were all important for determining the mode of dispersal in these species [107].

## Supporting information

S1 Fig Parsimony reconstruction of total axes length shows a gradual trend of axes reduction. See color legend in the figure corresponding to quantitative character states.
(TIF)

S2 Fig Parsimony reconstruction of pedicels length. See color legend in the figure corresponding to quantitative character states.
(TIF)

S3 Fig Boxplot of fruit length for DE, DE+IrA, IN, and IN+IrB modes of dehiscence (3 groups were removed as they had 3 taxon or less). No statistically significant differences were observed (p-value = 0.0825).
(TIF)

S1 Appendix List of herbarium vouchers used for the comparative study of inflorescence architecture. Species are arranged alphabetically. Country, locality details, date, collectors, and herbaria in which the specimens are deposited are provided for all specimens. Specimens used to determine pollen-ovule ratios are indicated with an asterisk (*). Herbarium acronyms are from Index Herbariorum [61].
(DOCX)

S2 Appendix Inflorescence, flower, pollen-ovule ratios and fruit characters. Infrageneric classification from [4]. Pollen-ovule ratio data from [15] except were indicated by an asterisk (*). Modes of dehiscence from [19]. IN = indehiscent; IrB = irregular type B; IrA = irregular type A; DE = dehiscent.
(DOCX)
