# Supplementary figures and images for: Inflorescences of Cuscuta (Convolvulaceae): Diversity, evolution and relationships with breeding systems and fruit dehiscence modes
Source: PLoS One. 2023 May 19;18(5):e0286100. doi: 10.1371/journal.pone.0286100 (PMC10198556; doi:10.1371/journal.pone.0286100)

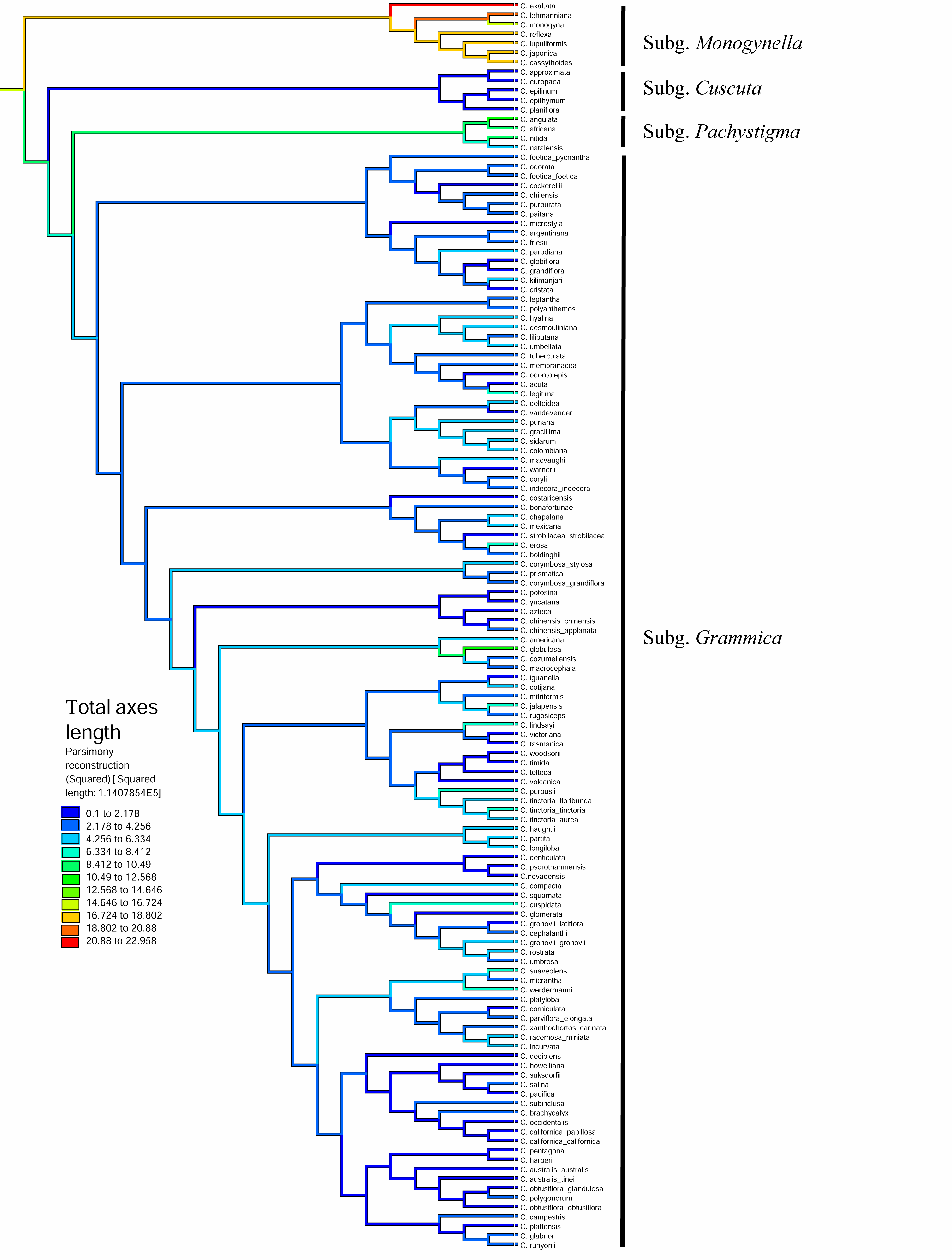

Supplement: S1 Fig — See color legend in the figure corresponding to quantitative character states. (TIF) [file pone.0286100.s001.tif]

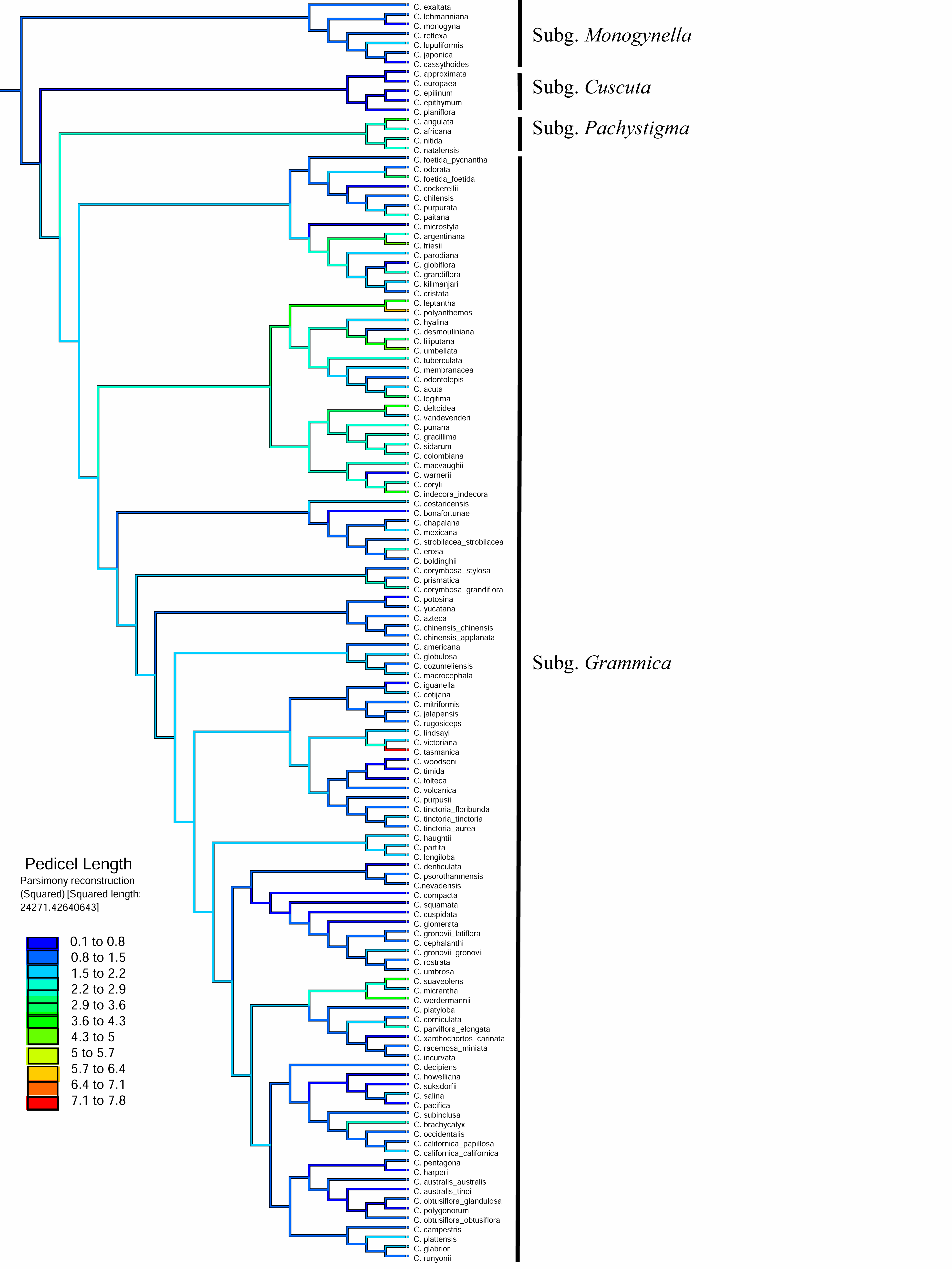

Supplement: S2 Fig — See color legend in the figure corresponding to quantitative character states. (TIF) [file pone.0286100.s002.tif]

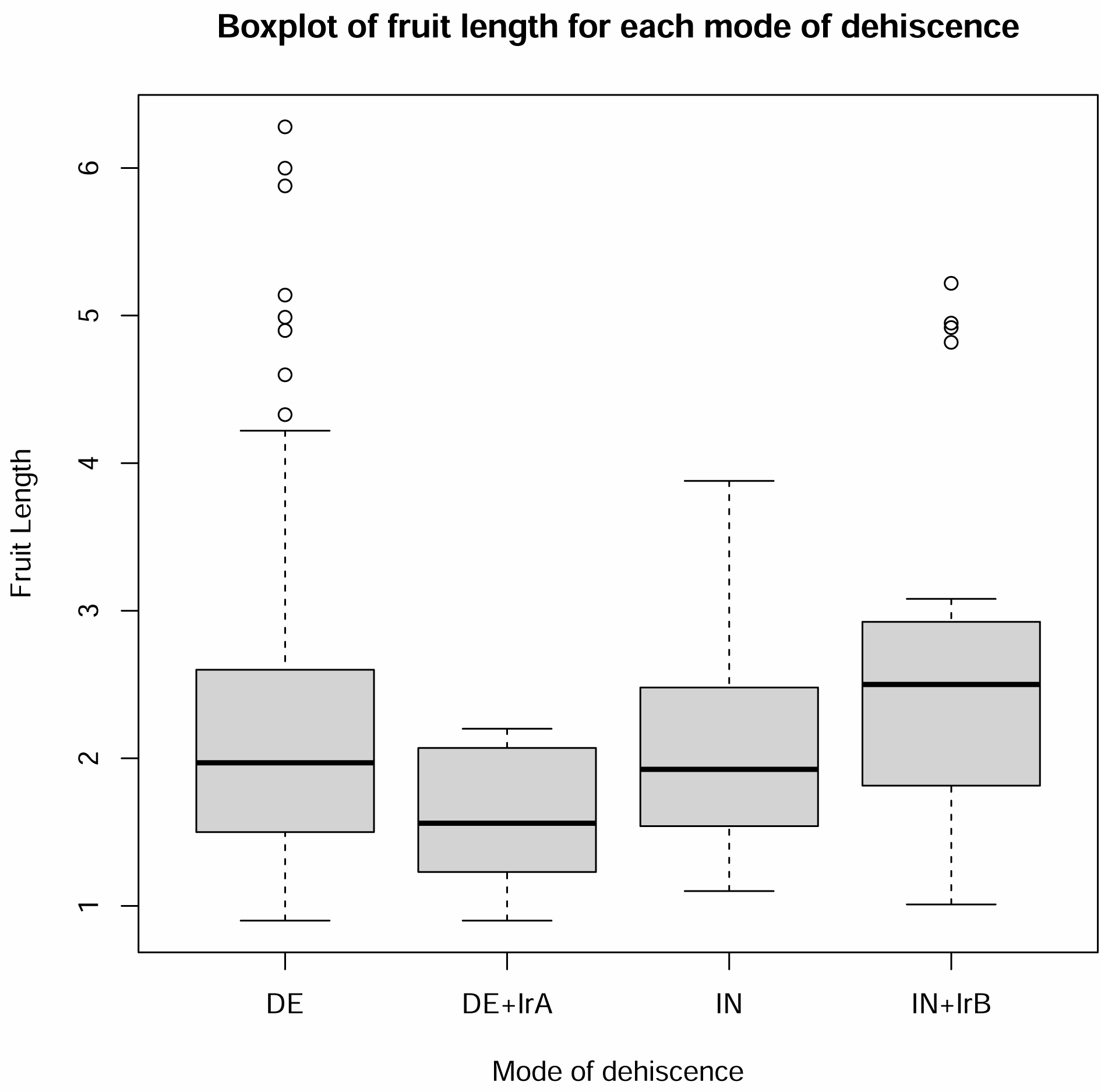

Supplement: S3 Fig — No statistically significant differences were observed (p-value = 0.0825). (TIF) [file pone.0286100.s003.tif]
