# Supplementary material for: Inflorescences of Cuscuta (Convolvulaceae): Diversity, evolution and relationships with breeding systems and fruit dehiscence modes
Source: PLoS One. 2023 May 19;18(5):e0286100. doi: 10.1371/journal.pone.0286100 (PMC10198556; doi:10.1371/journal.pone.0286100)
Supplement: S1 Appendix — Species are arranged alphabetically. Country, locality details, date, collectors, and herbaria in which the specimens are deposited are provided for all specimens. Specimens used to determine pollen-ovule ratios are indicated with an asterisk (*). Herbarium acronyms are from Index Herbariorum [61]. (DOCX) [file pone.0286100.s004.docx]

**Appendix S1.** List of herbarium vouchers used for the comparative study of inflorescence architecture. Species are arranged alphabetically. Country, locality details, date, collectors, and herbaria in which the specimens are deposited are provided for all specimens. Specimens used to determine pollen-ovule ratios are indicated with an asterisk (*). Herbarium acronyms are from Index Herbariorum.

***Cuscuta acuta*** Engelm.: **ECUADOR, Galapagos.** *Anderson 1853* (S); Tower Island, 16 Jun 1932, *Howell 10140* (G). **Manabí.** Bahia de Caraquez, Hotel La Herradura, 15 Feb 1981, *Benkt Sparre 19700* (S). ***C. africana*** Willd.: **SOUTH AFRICA.** *Muir 156* (GRA); *Oliver 11852* (NBG). **West Cape**, Kammanassie Mountains, in area Elands Vlakte 7, W side of Kammanassieberg, W facing slope, 11 Jan 2001, *Oudtshoorn et al. 11852* (NBG). ***C. americana*** L.: **MEXICO, Sonora,** Alamos, Arroyo el Mentidero at El Chinal Rd, 11.3 km S of Alamos, 26^o^54'45"N, 108^o^55'05"W, 240 m, 15 Mar 1994, *Van Devender 94-176* (UCR). **Guerrero.** Acapulco and vicinity, Oct 1894, Mar 1895, *Palmer 341* (GH). **Jalisco.** Mpio. La Huerta, near Chamela, 15 Apr 1982, *Lott 994* (MICH). **Sonora.** Mpio. Villa Pesqueira, S foot of Sierra Pinta, about 33 Mi S Moctezuma, ca. 29^o^35'N, 110^o^01'W, 15 Sep 1996, *Shortman 96-71* (ARIZ). **U.S.A., Florida.** Lower Matecumbe Key, 3 Jan 1925, *Small et al. 11596* (NY); Pinelands, Buena Vista, Miami, Jan 1930, *Mosier s.n* (NY). ***C. angulata*** Engelm.: **SOUTH AFRICA.** Near Farm “Alwee,” on road from Swellendam to Infanta, N slope of Pottberg in an area of sandy soil and shale chips, 24 Sep 1973, *Carlquist 4691* (RSA); *Beyers 12-1985* (NBG); *Williams 2690* (NBG). ***C. approximata*** Bab.: **CANADA, British Columbia.** Cherry Creek, Kamloops Lake, 30 Jul 1993, *Lomer 93-204* (UBC); Spences Bridge, 27 Sep 1992, *Lomer 92-306* (UBC). **U.S.A., California.** *Abrams 457* (CAS). **Nevada.** *Hillman s.n* (RSA). **Utah.** Salt Lake City, 24 Sep 1905, 4300ft, *Jones s.n* (RSA). ***C. argentinana*** Yunck.: **ARGENTINA, Corrientes.** Quebrada de Escoipe, Malcanto, 1940 m, Ruta 33, 10 Apr 1980, *Krapovickas & Schinini 36049* (CTES). **Salta.** Chicoana, La Zanja, 10 km N de la Quebrada de Escoipe (Ruta 33), 2700 m, 10 Apr 1980, *Krapovickas & Schinini 36757* (CTES). ***C. australis*** var. ***australis*** R. Br.: **AUSTRALIA, New South Wales.** Warrabah National Park, flood area for Namoi River, 30^o^34'S, 150^o^55'E, 425m, 13 Mar 1994, *J. R. Hosking 938* (CANB). **Queensland.** Maryborough, Wallum not far from sea, 23 Oct 1948, *Clemens s.n* (RSA). **CHINA, Guangxi.** Near Guilin, Yangshan, Guangxi Institute of Botany, 2 Sep 1988, *Sykes CH99* (CHR). **NEW CALEDONIA.** Nouméa, Feb 1907, *Bonati 737* (S). ***C. australis var. tinei*** (Insenga) Yunck.: **HUNGARY.** Tisza-Koff. *Simonkai 2635* (NY). ***C. azteca*** Costea & Stefanović: **MEXICO, Federal District**, Mexico City, Pedrigal, Valley of Mexico, 8 Oct 1896, *Pringle 6575* (S). **Puebla.** 18^o^10'48"N, 97^o^27'00"W, 9 Jul 1908, *Purpus 3554* (MO). **Querétaro.** 13.8 km SE MEX 120 on the road to Bucareli, Mpio. Piñal de Amóles, *Ramírez-Amezcua et al. 1099* (IEB). **San Luis Potosi.** Charcas, Jul - Aug 1934, *Lundell 5193* (S). ***C. boldinghii*** Urb.: **HAITI.** Massif de la Hotte, close to Dame Marie, on the beach, 1 Aug 1928, *Ekman 10455* (S). **MEXICO, Jalisco.** 8 km E of Chamela, 30-50m, lowland forest, 8-10 Dec 1970, *McVaugh 25140* (MICH). ***C. bonafortunae*** Costea & I. García: **MEXICO, Guanajuato.** Santiago de Maravatío, close to Ojo de Agua, 1900 m, 15 Oct 1989, *Rzedowski 49127** (IEB). **Michoacán.** Zamora, 11 Sep 2010, *García Ruiz & García 8375** (CIMI, WLU); 16 Oct 2010, *García Ruiz 8391** (CIMI, WLU). ***C. brachycalyx*** (Yunck.) Yunck.: **U.S.A., California.** Butte Co., west of Lunt Road, about 1/2 mile NW of Highway 70, 1800 ft, 10 Aug 1988, *Ahart 6170* (CAS); Kern Co., 1.5 mi N of Kernville, 2670 ft, 28 Sep 1962, *Howell 38877* (CAS); Santa Clara Co., between Sunnyvale and Alviso, lower end of San Francisco Bay, 22 Aug 1933, *Keck 2473* (CAS). ***C. californica*** Hook. & Arn. var. ***californica*** **U.S.A., California.** Colusa Co., dry slopes along upper reaches of Dark Hollow Creek, Snow Mt., 6600 ft, 10 Sep 1974, *Heckard 3888* (JEPS); Los Angeles Co., ridge S of Swartout Valley, 8200 ft, 31 Aug 1923, *Munz 7689* (RSA); Coastal Sage Scrub, Wash outside RSABG, 9 Oct 1965, *Massey 1005* (SMU). ***C. californica*** Hook. & Arn. var. ***papillosa*** Yunck..: **U.S.A., California.** Riverside Co., Colorado Desert, Coachella Valley, 33°53'N 116°15'W, 20 Apr 1995, *Sanders 16587** (UCR); Pushawalla Wash, 7 Dec 1946, *Roos 3184** (UCR); San Bernardino Co., Devil Canyon, 12 Jun 1987, *Sanders 7125** (UCR); Union Flat, 28 Aug 1978, *Krantz s.n* (UCR). ***C. campestris*** Yunck.: **U.S.A., California.** San Bernardino Co., near pond, North Verde Ranch, near the Mojave River E of Victorville, 2800 ft, 10 Sep 1961, *Raven 16637* (RSA); San Bernardino Mountains, Los Rios Rancho at Oak Glen Forest Falls, 4800 ft, 11 Nov 1999, *Sanders 23249* (UCR). **Maryland.** Wicomico Co., 4 Sep 1942, *Moldenke 13847* (OSU). **Florida.** Dade Co., 14000 SW 8^th^ Road, Miami, 12 Nov 1974, *Corell 43759* (NY). **Utah.** Salt Lake Co., Wasatch Range ca. 9 ½ mi. E of Salt Lake City, 5 mi. E of mouth of Emigration Canyon, 5600 ft, 1 Sep 1975, *Arnow 4694* (NY); Wayne Co., Caineville, 4500 ft, 20 Jul 1894, *Jones 5653* (RSA). ***C. cassythoides*** Nees ex Engelm.: **SOUTH AFRICA, Zululand.** Mtunzini, ‘Twinstreams’ Farm, Apr 1977, *Garland s.n.* (NU); *Drege s.n.* (MO). ***C. cephalanthi*** Engelm.: **U.S.A., Indiana.** Newton Co., Near the Kankakee River N of Lake Village, 22 Sep 1927, *Deam 455* (NY); Sullivan Co., Along a dredged ditch 6 mi SW of Carlisle, 4 Oct 1931, *Deam 51439* (IND). **Michigan.** Wayne Co., Oakwood, 15 Sep 1918, *Farwell 5124* (NY)*.* **Minnesota.** St. Louis Co., Low shore ledges of Sand Point Lake at Harrison’s Narrows international boundary, 11 Sep 1952, *Lakela 15846* (DAO)*.* **Utah.** Salt Lake Co., Salt Lake City, 4300 ft., 22 Aug 1880, *Jones s.n* (RSA). ***C. chapalana*** Yunck.: **MEXICO, Jalisco.** Rancho “Las Papas de Arriba”, 4.5 km NE of Guadalupe Victoria, 21°43′48″N, 101°39′48″W, 2260 m, 14 Oct 2000, *García & Harker 438* (IBUG); Mpio. Jocotepec: N face of Cerro Viejo, 2200 m, 17 January 1987, *Chazaro et al. 4408* (IBUG, XAL); Barranca de Sayula, al SE de San Pedro Tesistan, Nov 1993, *Machuca 7026* (MICH). ***C. chilensis*** Ker Gawl. **CHILE, Región de Coquimbo**, Prov. Elqui, Punta de Teatinos, Coquimbo Bay, 20 m, 21 Nov 1935, *West 3924* (MO). **Región Metropolitana**, Lo Barnechea, hills near Nido de Aguilas school, 33°30’S, 70°30’W, 550 m, 21 Jan 1978, *Landrum 3073* (ASU, MO)**. Valparaíso.** Uspallata-Pass der chilenischen Hochcordillere, Juncal, auf den Bergen, 16 Jan 1903, *Buchtien s.n* (UPS). ***C. chinensis*** Lam. var. ***applanata*** (Englm.) Costea and Stefanović: **MEXICO, Chihuahua.** Santa Eulalia, Buena Tierra Mine, ca. 28^o^37’N, 105^o^53’W, 6200 ft, 21 Aug 1948, *Hewitt 323* (GH). **Coahuila.** Near tip of Sierra de San Marcos, *Lewis s.n* (NY); Western Coahuila near Rosario, a railroad station 45 km W of Cuatro Cienagas, ca. 1100 m, 3-4 Sep 1941, *Johnston 8826* (GH); ca. 25 mi E of Americanos, 22 Aug 1937, *Wynd 754* (NY). **U.S.A., New Mexico.** Albuquerque, 28 Sep 1932, *Casteller 7339* (UNM). ***C. chinensis*** Lam. var. ***chinensis***: **AUSTRALIA.** Western Australia, at NE end of airstrip One Arm Point, N Dampier Peninsula, W Kimberley, 16^o^27’S, 123^o^04’E, 9 Apr 1993, *Carter 628* (CANB, PERTH). **CHINA, Shantung.** Tsinanfu, 4 Sep 1930, *Chiao 3033* (GH, NY). **NORTH KOREA.** Rikiho, Sep 1930, *Dorsett & Morse 6336* (NY). ***C. cockerellii*** Yunck.: **ARGENTINA.** *Vargas 2600* (CUS); *Vargas 19383* (CUS); *Nunez 28* (USM). ***C. colombiana*** Yunck.: **COLOMBIA, Magdalena.** Near Riohacha, 30 m, 23 Dec 1944, *Haught 4535** (KEW, S). **VENEZUELA,** Guarico: Parque Lazo Marti, La Aguada, Calabozo, Mar 1961, *Aristeguieta* 4568* (F); Feb 1961, *Aristeguieta 4500** (VEN). ***C. compacta*** Juss. ex Choisy: **U.S.A., Alabama.** Dekalb Co., Westfork Branch, De Soto State Park, Fort Payne, 850 ft, 13 Sep 1962, *Demaree 46247* (NY). **Arkansas.** Pulaski Co., Des Moulins, 27 Sep 1931, *Demaree 8260* (NY). **Florida.** Alachua Co., along Hawthorn Rd. ca. 4 mi E of Gainesville, 20 Nov 1943, *Rhoads & West s.n* (NY). **Indiana.** Crawford Co., on Pilot knob, about 4 mi S of Marengo, 2 Sep 1937, *Deam 58335* (NY); Posey Co., ½ mi S of Half Moon Pond which is 10 mi SW of Mt. Vernon, 26 Sep 1920, *Deam 33,043* (NY). **Oklahoma.** McCurtain Co., 3 mi N of Broken Bow, 13 Oct 1957, *Waterfall 14770* (RSA). ***C. corniculata*** Engelm.: **BRAZIL, Bahia.** Piatá, Gerais da Inúbia, 22-26 km de Catolés, 1400 m, 10 Mar 1992, *Stannard et al. H51861* (G). **COLOMBIA.** Intendencia Meta Villavicencio, 450 m, 26-31 Aug 1917, *Pennell 1453* (GH). ***C. coryli*** Engelm.: **CANADA, Manitoba.** District de Provencher entre Saint-Pierre et Otterburne, 27 Aug 1956, *Bernard 5669* (DAO). **Saskatchewan.** Ravine S of Qu’Appelle Valley 4 m N of Stoney Beach, 20 m NE of Moose Jaw, *Ledingham 3160* (USAS). **U.S.A., Indiana.** Perry Co., top of wooded flood plain bank of the Ohio River just below Derby, 11 Oct 1931, *Deam 51589* (IND). **Missouri.** Barry Co., Eagle Rock, 28 Sep 1896, *Bush 202* (MO, NY). **New York.** Tioga Co., Long Island, sea cliff, 24 Sep 1928, *Ferguson 7181* (NY). **West Virginia.** Ohio Co., on bank of Ohio River just below Eight Street, Wheeling, 16 Sep 1951, *Bartholomew 0-923* (NY). ***C. corymbosa*** Ruiz & Pav. var*.* ***grandiflora*** Engelm.: **MEXICO, Baja California Sur.** Gulf of California, isla Partita, 22 Apr 1921, *Johnston 3222* (GH). **Jalisco.** Sierra de Manantlán Occidental W facing slopes of Arroyo Las Joyas, 19^o^35’15”, 45”N, 104^o^15’30”, 45”W, 1 Jan 1984, *Iltis & Guzman 29077* (MICH). **Tamascaltepec.** Cumbre-Cruz, Alnus woods, 15 Mar 1936, *Hinton et al. 8984* (GH). ***C. corymbosa*** Ruiz & Pav. var*.* ***stylosa*** Engelm.: **MEXICO, Hidalgo.** Mpio. San Salvador, km 135-137 on Laredo Hwy between Actopan and Ixmiquilpan, 8 Oct 1943, *Gilly & Cany 5* (MICH). **Mexico.** Vertiente sur del Cerro Tlapacoya, mpio. Chalco, 7 Nov 1971, *Rzedowski 28752* (MEXU). **Veracruz.** Zacualpan, Dec 1915, *Purpus 7564* (GH). ***C. costaricensis*** Yunck.: **MEXICO. Chihuahua.** Las Gallinas, +/- 15 km W of Yepachi, 28^o^27’ N, 108^o^31’W, 1500 m, 22 Oct 1984, *Levin 1440* (SD). **Durango.** El Saltito, Nombre de Dios, 13 Sep 1984, *Jienez & Acevedo 35* (MEXU); San José de Parrilla, +/- 4 km al W. 23^o^43’99” N, 104^o^8’99”W, 2150 m, 25 Oct 1983, *González & Acevedo 2758* (MEXU). **Jalisco.** Isla Alacranes (Chapala), Maleza, 1500 m, 17 Sep 1964, *Cota 77* (MEXU). ***C. cotijana*** Costea & I. García: **MEXICO, Colima.** Quesería, exit from Rd. Quesería to San Antonio, close to Montitlán, 1420 m, 14 Mar 2010, *García et al. 8337* (CIMI, WLU); N side of exit to El Carrizal from Rd. Quesería-San Antonio, 1300 m, 14 Mar 2010, *García et al. 8338* (CIMI, WLU). **Michoachán.** Cotija, Los Gallineros, ca. 1900 m, 19 Apr 1991, *Ruiz & Olmos 3289* (UCR) isotype. ***C. cozumeliensis*** Yunck.: **GUATEMALA.** *Kellerman 6589* (F). **MEXICO.** *Calzade & Nievea 9427* (XAL); *Vazquez 176* (MEXU). ***C. cristata*** Engelm.: **ARGENTINA, Buenos Aires.** Baradero, Estancia “Los Alamos”, Fundación Figueroa Salas, Final de la Reserva, 27 Oct 2003, *Robles 1511* (MO). **Córdoba.** Río Tereero, 10 Jan 1940, *Burkart 10399* (MO); *Lossen 314* (KEW). Rio III, entre Almafuerte y el Embalse del Rio III^2^, 27 Feb 1944, *Hunziker 4927* (S). **Corrientes.** Esquiina, Isla frente a Esquina, 30 Nov 1974, *Krapovickas et al. 26901* (G). **La Rioja.** Sañogasta, Villa Bustos, 10 Dec 1951, *Pentzell 19170* (SI). ***C. cuspidata*** Engelm.: **U.S.A., Indiana.** Posey Co., low fallow field along the Wabash River ca. 2 mi S of New Harmony, 24 Sep 1920, *Deam 33011* (IND, NY). **Kansas.** Meade Co., park, 2460 ft, 18 Sep 1944, *Horr E476* (BRIT, SMU). **Missouri.** St. Louis, 11 Aug 1891, *Eggert s.n* (CAS). **New Mexico.** Clayton, 24 Sep 1907, *Evans s.n* (NMC). **Texas.** Gray Co., 15 mi S of Lefors, Ste. 291, 28 Sep 1969, *Correll 38056* (CAS, NY); Robertson Co., SW ¼ Round Prairie Quad., U.S.G.S. 1966, Tx. Hwy. 7 and the Navasota River, 9 Oct 1981, *Starbuck 1198* (BRIT). ***C. decipiens*** Yunck.: **MEXICO, Zacatecas.** 89.6 road mi (144.5 km) NE of Hwy 45 along Hwy 54 to Saltillo, about 2 mi NW of highway opposite turnoff to Majoma, (25 mi S of San Tiburcio) then to the first gate in the fence (to right) into the flats near a small milpa, near 23^o^52’N, 101^o^44’W, 1950 m, 19 Oct 2001, *Henrickson 22781* (RSA). ***C. deltoidea*** Yunck.: **MEXICO, Michoacan.** Monte Leon, 11 Nov 1892, *Pringle 5350* (NMC). ***C. denticulata*** Engelm.: **U.S.A., California.** Inyo Co., steep canyon leading into Saline Valley from the S, 36^o^34’N 117^o^35’W, about 5000-6000 feet, 16 Sep 1960, *Thomas 8904* (RSA); Riverside Co., Morongo Wash at the San Bernardino Co. line, 2400 feet, 11 Oct 1932, *Wolf 4282* (BRIT); San Diego, Borego Valley, on Larres, 7 Apr 1940, *Howe 991* (SD). **Nevada.** Humboldt Co., Calico Mts., foothill area W of main Gerlach-Soldier Meadow Rd., 6 July 2000, *Tiehm 13319* (ASU). ***C. desmouliniana*** Yunck.: **MEXICO, Baja California**. Chavez Ranch airstrip, about 5 mi. NW of Mulegé, 22 Oct 1962, *Wiggins 18130* (MEXU). **Sonora.** Hills near Altar, 26 Aug 1884, *Pringle s.n* (NY); Low basaltic hills 15 mi S of La Palina, between Hermosillo & Guayanas, 2 Sep 1941, *Wiggins & Rollins 232* (GH); Hwy W from Hermosillo to Bahía Kino, 12 mi W of the Hwy Jct. in Hermosillo, 800 ft, 29 Jan 1963, *Dunn et al. 14130* (NY); At coast on N side of headland about 10 mi. due S of Desemboque, 22 Mar 1978, *Spellenberg 4943* (MEXU, NMC); Terrace above arroyo at mouth of Nacopuli Canon, SE end of Cerros Las perinolas, N of San Carlos, 28^o^01’N lat., 111^o^03’W long, 26 Apr 1985, *Burgess 6949* (MEXU). ***C. epilinum*** Weihe: **CANADA, Quebec.** Shefford, Saint-Alphonse, sur lin, ferme de M. Wilfrid Viau, 30 Jul 1941, *Cartier s.n* (DAO, QFA); St. Alphonse, 30 Jul 1941, *Barabe 16914* (BRIT, SMU); Sainte-Hélène, Kamouraska, 15 Aug 1942, *Cayouette s.n* (QUE). **“Lower Canada”.** Flax fields, 4 Aug 1880, *Pringle 204788* (CAS). **SWEDEN,** Angermanland, Paroecia Arnäs, Idbyn, 22 Aug 1937, *Samuelsson 1317* (RSA). ***C. epithymum*** (L.) L.: **CANADA, British Columbia.** Kootenay Lake, Balfour Bay, 16 Aug 1947, *Turner 5983* (ALTA). **MEXICO, Mexico.** Valley of Mexico, 7800 ft, 27 Jun 1901, *Pringle 8514* (NMC). **U.S.A., Idaho.** Custer Co,. Challis, edge of alfalfa field, 5400 ft, 15 Jul 1916, *Macbride & Payson 3219* (DS, RSA). ***C. erosa*** Yunck.: **MEXICO, Sonora.** 15 mi SE of Magdalena on rd. to Cucurpe, 11 Sep 1934, *Wiggins 7123* (NY); Agua Prieta, ca. 7.5 km SE of Agua 31^o^15’34”N, 109^o^36’34”W, 1233 m, 3 Oct 2004, *Van Devender et al. 2004-1199* (WLU). **U.S.A., Arizona.** Pima Co., Baboquivari Canyon, 3500-4500 ft., *Kearney & Peebles 10377* (CAS, RSA); *Kearney & Peebles 10422* (CAS, RSA); Baboquivari Mts, 21 Aug 1932, *Peebles 8998* (RSA); Baboquivari Mts, 19 Sep 1931, *Jones 28731* (CAS). ***C. europaea*** L.: **BELGIUM, Luxembourg.** Chassepierre, bord de la Semois en amont du village, 30 Sep 1975, *Duvigneaud & Lambinon 75B953* (QUE). **DENMARK, Dyrehaven.** Copenhagen, 6 Aug 1970, *Svendsen 329* (NY). **FINLAND, Kemiö**. Stenhol, 60^o^05’N, 22^o^45’E, Cliff near the old limestone quarries, 16 Aug 1978, *Alava et al. s.n* (NY, RSA). **GERMANY,** Alsfeld. Kestrich, Sengesweg, 12 Aug 1972, *Hupke s.n* (USAS). **Thüringen.** 20 Aug 1900, *Rudolph s.n* (RSA). **NETHERLANDS, Gelderland.** Base of levee in the Ooypolder E of Nijmegen, near Tiengeboden, 12 Sep 1959, *Hekking 635* (NY). **SWEDEN.** Blekinge Co., Sölvesborg, 23 Jul 1932, *Holmgren 19784* (SD). ***C. exaltata*** Engelm.: **U.S.A., Texas.** Aransas Co., Goose Island State Park, 1 Oct 1975, *Snyder 472* (SMU); Dallas, Sep 1885, *Reverchon 663* (CAS); Hays Co., Stagecoach Ranch subdivision off Ranch Road 3238, S of Hamilton Pool Preserve, 24 Sep 1992, *Westlund s.n* (CAS); Johnson Co., 97^o^36’N 32^o^15’W, Cleburne State Park, about 12 mi (19 km) WSW of Cleburne, incised valley along West Fork of Camp Creek at end of Park Road 21.6 mi (10 km) SW of US Hw 67, 27 Oct 1997, *Sanders 4270B* (BRIT); San Patricio Co., local, E edge of perimeter road on W side of installation, SW of jct. F. M. 2725 and F. M. 1069, Naval Station Ingleside, Port Ingleside Quadrangle, 25ft, 11 Sep 1992, *Carr 12341* (BRIT); Val Verde Co., Dry Devils River, E of crossing of road from “big house” N to mouth of Jane Hollow, Dolan Falls Ranch, Dolan Springs Quadrangle, 1530 ft, 30 Sep 1992, *Carr 12418* (SMU). ***C. foetida*** Hook. & Arn. var. ***foetida***: **ECUADOR, Azuay.** Laguna Llaviuco (Surucuchu) W of Cuenca, 3150 m, 25 Feb 1993, *Harling & Ståhl 26675* (S); **Canar.** Parroquia Bayas, Valley of rio Tabaca, ca. 15 mi NE Azugues, 8000 ft, 27 Sep 1944, *Prieto & Camp P-111* (S). **Chimborazo.** En el camio de Sibambe a Alausí, Reg. Interandina, 2500 m, 25 Aug 1943, *Solís 5599* (F). ***C. foetida*** Kunth var. ***pycnantha*** Yunck.: **ECUADOR, Cotopaxi.** Cantón Sigchos, Triunfo Grande, entrada a la comunidad “El Valle”, 2765 m, 00^o^34’02”S, 78^o^57’41”W, 31 Jul 2003, *Ramos et al. 6775* (MO). **Pichincha.** W of Nono, 2700 m, 12 Jun 1968, *Harling et al. 10258* (US). **PERU.** *Plowman et al. 14291* (F). ***C. friesii*** Yunck.: **ARGENTINA, Tucumán.** Infiernillo, 37 km de Tafí del Valle, 2650 m., 18 Mar 1972, *Krapovickas & Maruñak 21898* (CTES). ***C. glabrior*** (Engelm.) Yunck.: **MEXICO, Coahuila.** Parras de la Fuente, Sierra de Parras, Rancho El Tunal, 31.2 km (19.5 mi) al E de Parras por carretera, 4.8 km (3 mi) al S por terracería hasta la Puerta del sur del rancho, 1870 m, 21 Aug 1982, *Cowan 3644* (MEXU); ca 35 (air) mi SSW of Cuatro Cienegas, in northern slope of limestone Sierra de Los Alamitos, ca 9.2 (rd) mi S of El Hundido, in Izotal, 26^o^30’N, 102^o^17’W, 4650 ft,, 29 Sep 1973, *Henrickson 13676c* (RSA); Chojo Grande, 27 mi SE of Saltillo, 16 Jul 1905, *Palmer 723* (GH, MO). **U.S.A., Texas.** Deaf Smith Co., 15 mi N & 15 mi W of Hereford, 23 Jul 1966, *Waller 962* (TEX/LL). ***C. globiflora*** Engelm.: **ARGENTINA, Catamarca.** Andalgalá, El Condado, 25 Feb 1916, *Jorgeusen 1613* (SMU). **Jujuy.**  Tumbaya, ruta hacia Tilcara, Barcena, 1700 m, 14 Mar 1994, *Múlgura et al. 1199* (MO). **BOLIVIA.** Andian reg, Cotaña am Llimain, 2450 m, [no date], *Buchtien 133* (F). **Cinti.** Sucre, Puca Rhasa prope Tacaquira, 21-27 Mar 1934, *Hammarlund 341* (S). **Murillo.** La Paz, below Obrajes, ca 3300 m, 27 Jan 1921, *Asplund 2088* (S, UPS). ***C. globulosa*** Benth.: **CUBA,** Oriente, El Cobre in fructicetis communis, 6 Oct 1916, *Ekman 7839* (S). **PUERTO RICO, Culebra.** Playa Flamenco, at end of road along beach, 1 m, 15 Jul 1989, *Axelrod 1154** (UPRRP). **Guayama.** Rte 712, km 16.7, 1 km along farm track following ridge S of road, ca. 350-400 m, 21 Jan 1991, *Axelrod 1875** (UPRRP). ***C. glomerata*** Choisy: **U.S.A., Indiana.** Lake Co., marsh 2 mi N of Hobart, 17 Sep 1930, *Deam 49868* (NY). **Kansas.** Riley Co., Horse Pasture, NW ¼ sec. 13, T 11 S, R 7 E, 10 Sep 1979, *Freeman 293* (NY). **Nebraska.**  Minden, Sep 1996, *Hapeman 21141* (NMC). ***C. gracillima*** Engelm.: **MEXICO, Michoacan**, 3km W of Ixtapan del Oro on rd. to Zitacuaro, 19^o^15’N, 100^o^16’W, 1900m, 3 Dec 1983, *Solheim et Benz 1073* (NY); *Hinton 2497* (F). **Sinaloa.** Mazatlan, approx. 23^o^12’N, 106^o^25’W, 0-30m, 20 Nov 1926, *Jones 22408* (UCR). ***C. grandiflora*** Kunth: **ECUADOR, Carchi.** Tulcan Canton, Colonia Huaqueña, Loma El Corazón, 77^o^42’W, 00^o^35’N, 3000 m, 8 Jul 1992, *Tipax et al. 1563* (QCNE, MO); Ca. 2 km along the road El Angel-Tulcán, hedges along the road, 77^o^55’W, 0^o^38’N, 3150-3300 m, 14 May 1973, *Holm-Nielsen et al. 5214* (AAU); Km 3 on old road El Angel-Tulcan, 77^o^55’W, 00^o^39’N, 3200 m, 13 Aug 1985, *Laegaard 54907A* (QCA). **PERU, Cuzco.** Cuzco, Mar 1929, *Herrera 2354a* (F). **Huerta.** Conima, 3900 m, 6 Mar 1948, *Aguilar s.n.* (MO). **Paucartambo.** Cusco, along Río Paucartambo, S of Paucartambo, 13^o^18’S, 71^o^40’W, 3 Oct 1995, *Croat 78149* (BRIT, MO). **San Sebastián.** Cusco, 3300-3400 m, 25 Apr 1925, *Pennell 13613* (F). ***C. gronovii*** var. ***gronovii*** Willd. ex Schultes: **CANADA, Quebec.** Cté de Chauveau, St-Augustin, face au Séminaire, 14 Aug 1975, *Pérusse 75-39*1 (QFA); Comté de Lotbinière, Sainte-Croix-de-Lotbinière, 2 km au sud-est de la Pointe au Platon, 46^o^39’10”N, 71^o^49’30”W, 24 Oct 1989, *Garneau et Roy 89-626-M* (QFA); Ottawa Distr. Near Gatineau Point, 2 mi N of Ottawa, 14 Sep 1952, *Dore & Erskine 14109* (DAO). **New Brunswick.** Kent Co., St. Louis Parish, 2.6 mi into Kouchibouguac National Park on Cap St. Louis Rd. along shore past gate, 9 Sep 1977, *Munro & Cody 1786* (DAO). **Ontario.** near Long Point Provincial Park, 42°35'43.34"N, 80°27'2.66"W, 19 Oct 2014, *Costea & Ho s.n.* (WLU); Waterloo, Grand River, Claude Dubrick trail, 43°30'12.02"N, 80°29'37.97"W, 17 Oct 2014, *Costea & Ho s.n.* (WLU). ***C. gronovii*** var. ***latiflora*** Engelm.: **U.S.A., Indiana.** *Yatskievych 84-162* (IND). **Texas.** Wood Co., 5 mi NE of Crow, Lake Ellis, 2 Sep 1942, *Lundell 11721* (SMU). ***C. harperi*** Small: **U.S.A., Alabama.** Marion Co., North Fork Creek, S of Hackleburg on US 43, 7 Sep 1968, *Kral 32878* (SMU); [undated], *Damaree 46295* (NY). ***C. haughtii*** Yunck.: **ECUADOR, Guayas.** Salinas, La Puntilla, 6 Apr 1939, *Asplund 5618* (G, KEW, S). **Manabi.** Manta, 6 Apr 1955, *Asplund 15974* (G, S, UPS). ***C. howelliana*** Rubtzoff: **USA, California.** *True 6716* (CAS); Shasta Co., between Goose Valley and Burney Valley, ca. 3.5 mi NNW from Burney, 3200 ft, 8 Aug 1988, *Taylor 10026* (CAS). ***C. hyalina*** Roth.: **INDIA, Delhi.** Delhi University ridge, 8 Aug 1954, *Mkhanno 249* (ARIZ); Delhi University campus, 690 ft, 12 Feb 1961, *Pushpander s.n.* (CANB). **South West Africa.** Mar 1931, *Bosch 25022* (BOLUS). ***C. iguanella*** Costea & I. García: **MEXICO, Jalisco.** Wooded hills near Guadalajara, 2 Sep 1893, *Pringle 4529* (F, GH, MEXU, S). ***C. incurvata*** Progel: **PARAGUAY,** North Paraguay**.** In regione cursus superioris fluminis Apa, Dec 1901, *Hassler 8178* (F); Zwischen Rio Apa und Aquidaban, 1908/1909, *Fiebrig 5083* (K). **Caaguazú.** 4 km al N de Yhú en cerrado, suelo arenoso, 6 Feb 2003, *López et al. 243* (CTES). ***C. indecora*** Choisy: **HONDURAS.** Malfredi Lagoon, 100 ft, 1 May 1933, *Schipp 1161* (S). **U.S.A., Arizona.** Gila Co., along roadside N of Winkelman, S of Globe, 5000 ft, 16 Aug 1973, *Moldenke 27920* (AAU); Pinal Co., near Peppersauce Campground on N side of Santa Catalina Mountains, ca. 15 mi SE of Oracle, 4700 ft, 30 Aug 1989, *Austin 7599* (RSA). **Nebraska.** Arthur Co., Arapaho Prairie, T18N R39W Sect 31, 32, 1200 m, 27 Jul 1977, *Vescio & Kruse 174* (NY). **Nevada.** Nye Co., U.S. Atomic energy commission’s Nevada test site and vicinity, Abandoned field, Rt 52 near Rt 16 jnct., Cent. Pahrump, 2600 ft, 26 Sep 1970, *Beatley s.n.* (RSA). **New Mexico.** *Spellenberg et al. 3427* (NY). **Utah.** Salt Lake City, 4200 ft, 25 Aug 1879, *Jones s.n.* (RSA). ***C. jalapensis*** Schlecht.: **GUATEMALA, Totonicapán.** Region of Chui-quisís, above Totonicapán on Rd. to Desconsuelo, 2500-2800 m, 23 Jan 1948, *Standley 84397* (NY). **MEXICO, Chiapas.**  Amatenango del Valle, 5800 ft, 26 Jul 1966, *Breedlove 14669* (MICH); San Cristóbal de Las Casas, Santa Cruz in San Felipe, 15 Nov 1986, *Ton & Lopez 9826* (GH); Tenejapa, Paraje Shohleh, 2560 m, 12 Jan 1966, *Ton 603* (NY). **Hidalgo.** El Chico, alrededores de Peña del Cuervo, 5 km al SE de El Chico, 2800 m, 20 Jul 1986, *Medina 3102* (MICH). **Puebla.** Near Huauchi-nango, 5000 ft, 27 Mar 1945, *Sharp 45380* (NY). ***C. japonica*** Choisy: **CHINA**, Bizen. 7 Oct 1925, *Masamune s.n.* (NY). **Guizhou.** Songtaoi Xian, vicinity of Lengjiaba in the vicinity of the confluence of the Xiaohe and Dahe rivers, NE side of the Fanjing Shan mountain range, 820-1120 m, 5-9 Oct 1986, *Bartholomew et al. 2309* (RSA). **Shaanxi.** Foping Co., 500 m, 16 Oct 1998, *Weiqing 619* (MO). **JAPAN, Hondo.** 17 Oct 1952, *Hashimoto 853* (NY). **Honshu.** Kyoto, Anshu, Yamashina-ku, Kyoto-shi, 70 m, 25 Oct 1998, *Tsugaru et al. 27202* (MO). ***C. killimanjari*** Oliv.: **MALAWI.** Lilongwe Nature Sanctuary, 1050 m, near river, 29 Jun 1987, *LaCroix 4559* (MO). **ZIMBABWE.** *Eyles 352* (J). ***C. legitima*** Costea & Stefanović: **MEXICO, Baja California.** Along arroyo 3 ½ mi S of La Paz, ca. 24^o^09’N, 100^o^15’W, 3 Nov 1959, *Wiggins 15294* (MEXU). **Sonora.** Cerro La Antena, 1 km N of Microondas La Cabana; Sinaloan thornscrub, 27^o^27’45”N, 109^o^46’20”W, 200 m, 19 Sep 1994, *Van Devender 94-603* (ARIZ). **U.S.A., Arizona.** Douglas, 11 Sep 1948, *Jones s.n.* (RSA). ***C. lehmanniana*** Bunge: **KAZAKHSTAN.** Turkestan, 12 Feb 1910, *Fedtschenko 1* (NY). **UZBEKISTAN.** Samarkand, 30 Jul 1914, *Knorring 104* (NY); Syr-Darja, Tashkent, 1 Aug 1924, *Vvedensky 153* (NY). ***C. leptantha*** Engelm.: **MEXICO, Baja California.** 10 mi inland from Bahia de Los Angeles near Agua Armaga, near 29^o^00’N, 113^o^45’W, 22 May 1960, *Lindsay 2928* (SD); On arid hills near Club Aereo airport, Mulegé, 9 Apr 1963, *Wiggins & Wiggins 18219* (K); Bahia de Los Angeles, SE shore, near intersection of a road leading to the beach, along roadside, 14 Mar 1992, *Fritsch & Fritsch 1337* (RSA). ***C. liliputana*** Costea and Stefanović **U.S.A**. **Arizona**, Pima Co.: ca 15 mi SE of Tucson, along Haughton Rd., 1 mi N of I10, 731 m, 20 Oct 1982, *Neese s.n** (NY). **New Mexico**. De Baca Co.: Hwy 20, just S of Conejo Creek, ca. 24 mi SW of Fort Sumner, T1S R24E Sec 29 Ne1/4, 1250 m, 26 Sep 2002, *Sivinski 5689** (NMC, NY, TEX). Doňa Ana Co.: White Sands Missile Range, 29 Km NNE of las Cruces, 3 Km S of US Hwy 70, W edge Sec 7, T22S, RSE; UTM 360900/3586500, 1300 m, 27 Aug 1990, *Spellenberg & Brozka 10526** (NMC, ID, UC). Sierra Co.: 3 mi S of Hillsboro, 1680 m, 9 Sep 1904, *Metcalfe 1290** (ARIZ, MO, NY, SD, UNM, WLU). ***C. lindsayi*** Wiggins: **MEXICO, Sinaloa.** On talus slope 55.7 mi E of Villa Union, 6210 ft, 18 Mar 1955, *Wiggins 13185* (MO). ***C. longiloba*** Yunck.: **BOLIVIA, Azero.** Chuquisaca, Estsoión Experimental Zootecnica “El Salvador (27 km NW de Carandaytí, 63^o^13’W, 20^o^45’S, 500 m, 800 m NE de la entrada a la Est. Exper., 13 Apr 1977, *Krapovickas & Schinini 31255* (CTES). **PARAGUAY, Chaco.** Parque Nacional Defensores del Chaco, a 30 km de Aguarrica en dirección a Lagerenza, 21 Oct 1980, *Casas & Molero s.n.* (MO). ***C. lupuliformis*** Krock.: **AUSTRIA.** Ca. 1.2-1.25 km SE Baumgarten an der March, 14 Aug 2004, *Barta 2004-177* (NY); ca. 0.25 km ENE Sierndorf an der March, 28 Jul 2004, *Barta 2004-302* (NY). **HUNGARY.** Prope Budam, 18 Aug 1898, *Degen s.n.* (RSA). ***C. macrocephala*** Schaffner: **MEXICO, Baja California.** Along road to El Valle Perdido 4 mi. E of La Paz-Todos Santos road, about 23^o^43’N, 110^o^10’W, 350 m, 6 Nov 1959, *Wiggins 15323* (K). **Sinaloa.** Cosalá, 24°24'16"N, 106°41'26"W, 4 Jan 2006, *Van Devender 2006-15* (WLU); *Van Devender 2006-16* (WLU). **Sonora.** Álamos, 27°06'34"N, 108°42'58"W, 5 Oct 2006, *Van Devender 2006-1240* (WLU); Arivechi, 28°49'15"N, 109°10'24"W, 15 Sep 2006, *Van Devender 2006-872* (WLU); San Javier, 28°34'48"N, 109°40'15"W, 31 Aug 2001, *Van Devender 2001-758* (WLU); Yécora, 28°25'48"N, 109°11'31"W, 1 Sep 2001, *Van Devender 2001-774* (WLU); 28°25'53"N, 109°09'34"W, 16 Sep 2006, *Van Devender 2006-901* (WLU); 28°20'50"N, 109°07'17"W, 21 Sep 1998, *Van Devender 98-1524* (WLU). ***C. macvaughii*** Yunck.: **MEXICO, Michoacán.** Churumuco, 18^o^37’09”N, 101^o^36’48”W, 8 Sep 2007, *Steinmann & Ramírez 5870* (IEB, WLU); Apatzingan, 19^o^01’40”N, 102^o^17’43”W, 13 Mar 2010, *García-Ruiz & Alvarez 8335* (CIMI, WLU). ***C. membranacea*** Yunck.**, Argentina**, **Provincia de la Rioja**, Depto. de Capital, La Ramadita, 25 Km of Rioja, 17 Feb 1944, *Hunziker 4823* (S, US); 17 Feb 1944, *Hunziker 4833** (S, US); 9 Mar 1944, *Hunziker 4695** (S). Provincia La Salta, Depto. de la Pima, 2 Feb 1941, *Hunziker1149** (NY). ***C. mexicana*** Yunck.: **MEXICO, Jalisco.** Autlan, 16 May 1990, *Cuevas & Núñez 3834* (IEB, ZEA). ***C. micrantha*** Choisy: **CHILE, Atacama.** Llano de Churque, S de Copiapó, 27^o^38’S, 70^o^28’W, 7 Oct 1987, *Teillier 894* (MO); Huasco, Isla Guacolda, 5-15 m, 26 Oct 1938, *Worth & Morrison 16235* (MO). ***C. microstyla*** Engelm.: **ARGENTINA.** *Boelcke et al. 10243* (CTES). ***C. mitriformis*** Engelm.: **MEXICO, Chihuahua.** Sierra Madre, 2 Oct 1887, *Pringle 1342* (MO). **Coahuila.** Ca 22 (air) mi WNW of Cuatro Cienegas, in upper portion of limestone Canyon de la Hacienda, below 1^st^ lumber camp in Sierra de la Madera, 5000-6000 ft, near 27^o^04’N, 102^o^25’W, 28 Sep 1973, *Henrickson 13638* (RSA). **Michoacan.** Mountains near Lake Chapala, 18 Nov 1892, *Pringle 4330* (S). **Nuevo Leon.** Hacienda Pablillo, Galeana, 8 Jan 1936, *Taylor 38* (F). ***C. monogyna*** Vahl.: **CANADA, Ontario.** Mississauga, grown in greenhouse at U of T, 13 Jul 2015, *Stefanović s.n.* (TRTE, WLU). **GREECE, Lihadha.** Istiea, part of the island of Euboea, 1-2 m, 29 July 1973, *Greuter 11459* (OSU). **UZBEKISTAN, Andijan.** 25 Jul 1911, *Knorring & Minkwitz 1590* (NY). ***C. natalensis*** Baker: **SOUTH AFRICA, Natal**, *Rudatis s.n.* (NBG). ***C. nevadensis*** I. M. Johnston: **U.S.A., California.** Inyo Co., lower end of Westgaard Grade near Big Pine, 5400 ft, 10 Jul 1938, *Jaegar s.n.* (RSA). **Nevada.** Rye Co., sands W of Lathrop Wells, 2500 ft, Amargosa drainage basin, 19 Jun 1969, *Beatley s.n.* (RSA). ***C. nitida*** Meyer: **SOUTH AFRICA.** At memorial along contour trail to summit of 11:30 Peak, Clock Peaks, near Swellendam, 2000 ft, 11 Dec 1973, *Carlquist 5082* (RSA); *Compton 15500* (NBG), *Taylor s.n.* (NBG). ***C. obtusiflora*** H.B.K. var. ***glandulosa*** Engelm.: **MEXICO, Tamaulipas.** At edge of lake, 600 ft, 24 Jul 1939, *Chase 7563* (MO). **U.S.A., Texas.** Dallas, [undated], *Reverchan 2878* (MO); Dallas Co., bordering lake, Bachmans Dam, 24 Aug 1944, *Lundell 11626* (SD); Rio Grande, 1848, *Wright s.n.* (MO); Bastrop Co., Sep 1937, *Tharp s.n.* (MO); Austin, 14 Aug 1934, *Tharp s.n.* (UC1). ***C. obtusiflora*** var. ***obtusiflora*** Kunth: **ARGENTINA, Corrientes.** Concepción, 21 Dec 1977, *Tressens & Sesa 12026* (MO); near San Cosme, 29 Jan 1970, *Pedersen 9628* (MO); Ituzaingó, Isla Apipé Grande, huerto San Antonio, 19 Nov 1976, *Guaglianone et al. 138* (SI). **U.S.A., Delaware.** Bank of creek, Middletown, 6 Aug 1911, *Churchill 672* (MO). ***C. occidentalis*** Millspaugh: **U.S.A., California.** Los Angeles Co., San Clemente Island, Sand dunes of coastal terrace at West Cove, SW of the new landing field, 20 feet, 17 May 1991, *Ross et al. 5087* (RSA); Marin Co., Mt. Tamalpacs, 1200 ft, 8 May 1922, *Munz 6445* (RSA); Siskiyou Co., Siskiyou Mountains, Lily Pad Lake, 21 Aug 1958, *Wheeler 7417* (RSA). ***C. odontolepis*** Engelm.: **MEXICO.** Unknown locality, 1851-1852, *Wright 1624* (K). **Sonora.** Near a deserted Rancho on rocky hill sides, 15 Sep 1851, *Wright 529* (MO). ***C. odorata*** Ruiz & Pavon: **ECUADOR, Chimborazo.** Cañon of the río Chanchan near Huigra, 4000–4500 ft, 7-14 May 1945, *Camp 3027* (S). **PERU, Huarochiri.** Lima, San Mateo, 3200 m, 28 May 1940, *Asplund 11177* (S); Lima, along Rio Chillón, above Obrajillo, Open rocky slopes, 2800–3200 m, 13-23 Jun 1925, *Pennell 14382* (S). ***C. pacifica*** Costea & M. A. R. Wright: **U.S.A., California.** Humboldt Co., Humboldt Bay near Table Bluff, 28 Aug 1941, *Harris 1175* (B); Santa Cruz, 30 Jun 1881, *Jones 13467* (MO); Thorne, 8 Aug 1965, *Gveaelt 75280* (UC). ***C. paitana*** Yunck.: **PERU, Paita.** Piura, 150 m, 16-17 Mar 1927, *Weberbauer 7762* (F); Pariñas Valley about 6 mi inland, 26 Dec 1928, *Haught F-100* (F). ***C. parodiana*** Yunck.: **ARGENTINA, La Salta.** La Caldera, Yacones, Laderas de cerros al W del camino y rio, 1700 m, 29 Apr 1990, *Novara & Bruno 9821* (S). **Jujuy.** Ledesma, camino de Fraile pintado a El Aibal, 13 Nov 1992, *Kiesling 8236* (MO); locality illegible, 26 Feb 1901, *Kurtz 11792* (S). ***C. partita*** Choisy: **BRAZIL, Maranhão.** Lorêto, Ilha de Balsas region, between the Balsas & Parnaíba Rivers, About 35 km S of Lorêto, 100 m NE of main house of Fazenda Morros, 7^o^23’S, 45^o^4’W, 300 m, 3 Apr 1962, *Eiten & Eiten 3961* (US). **Piaui.** Corrente. BR-135, 500 m S da ponte sobre o rio Corrente, 10^o^27’S, 45^o^9’W, 460 m, monte abierto, 3 Apr 1983, *Krapovickas et al. 38723* (CTES). **COLOMBIA.** Forest, Rincon Hondo, Magdalena Valley, 5 Aug 1924, *Allen 267* (F, MO). **VENEZUELA, Lara,** a orilla de la carretera Bobare-Aguada Grande 17 km antes del crusero a la ultima población, Edo. Lara, 29 Aug 1981, *Ponce & Trujillo 342* (OAC).  ***C. parviflora*** Engelm. var. ***elongata*** Engelm.: **BRAZIL, Goiás.** *Oliveira et al. 745* (US). ***C. pentagona*** Engelm.: **U.S.A., Florida.** Levy Co., Cedar Key, 10 May 1958, *Godfrey 56580* (NY). **Indiana.** Cass Co., about 1 ½ mi NW of Lake Cicott (P. O.), 1 Oct 1940, *Deam 60219* (IND); Starke Co., 2 ½ mi SE of North Judson, 18 Jul 1930, *Deam 49139* (IND). **Kansas.** Trego Co., 19 mi S and 2 mi W of Collier, 6 Aug 1952, *David & Harr 4136* (NY). **Massachusetts.** Tonset, 27 Aug 1901, *Edmondson 2777* (NY). **Michigan.** Kalamazoo Co., Fort Custer, 12 Aug 1945, *Hanes 4541* (NY). **Texas.** Hunt Co., 7.4 mi E of Greenville, 8 Jun 1953, *Shinners 15030* (TEX/LL). **Virginia.** Beoford Co., [no date], *Curtiss s.n* (NY). ***C. planiflora*** Ten.: **AUSTRALIA.** Unknown locality, unknown date, *Easkins s.n.* (WLU). **PALESTINA.** near village of Kesan, near Tekoa, 31^o^35’N, 35^o^15’E, 22 May 1987, *Musselman 10461* (RSA). Unknown locality, undated, *Priva 82* (S). ***C. plattensis*** A. Nelson: **U.S.A., Wyoming.** Goshen Co., T25N R60W Sec 31 S ½ SW ¼, 3 mi. NE of Torrington, 4200 ft, 15 Aug 1993, *Dorn 5470* (MO); Platte Canon, 27 Aug 1896, *Nelson 2741* (MO). ***C. platyloba*** Progel: **ARGENTINA, Misiones.** San Ignacio, Balneario Teyú Cuaré, 27^o^16’S, 55^o^35’W, 23 Nov 1995, *Guaglianone et al. 3025* (K). **BRAZIL, Rio Grande Do Sul.** Osório, 3 m, 19 Jan 1951, *Sehnem 5597* (B). **COLOMBIA.** Intendencia meta Villavicencio, grassy plains E of V, 450 m, 26-31 Aug 1917, *Pennell 1453* (MO). ***C. polygonorum*** Engelm. **CANADA**, **Quebec**, MRC Beauharnois-Salaberry, Îles de la Paix, Île à Tambault (Station 2): berge, 4 Sep 1965, *M. Morency 1651** (MT).Vaudreuil-Soulanges, Île-Perrot, près de la Pointe-du-Moulin. Rivages graveleux et rocheux, 5 Sep 2005, *Hay* & *C. Morisset SH05-189** (MT), *SH05-190** (MT), *SH05-191** (MT). **U.S.A.** **Arkansas** St. Francise Co.: 5 mi S of Forrest City, 19 Sep 1959, McDaniel 1419 (NY). **Indiana**. Putnam Co.: 2 mi E of Bainbridge, 20 Oct 1941, *Yunck. 10836* (NY). **Nebraska**. Otoe Co.: extreme S part of SE1/4, NE1/4, Sec 25, T8N-R14E, 11 Sep 1974, *Sutherland 4096* (NY). ***C. polyanthemos*** Schaffner ex Yunck.: **MEXICO, Sonora.** Monctezuma, 29^o^39’44”N, 109^o^37’13”W, 14 Sep 2006, *Van Devender 2006-809* (WLU); 31 mi NW Queriego, 6 Mar 1933, *Wiggins 6457* (US). ***C. potosina*** Schaffner: **MEXICO, Estado de Mexico.** Pedrigal (lava beds), Valley of Mexico, 7300 ft, 8 Oct 1896, *Pringle 6575* (MO). **Guanajuato.** Mpio. De San Diego de la Unión, 11 Sep 1997, *Pérez et al. 3707* (IEB). ***C. prismatica*** Pav. ex Choisy: **ECUADOR, Guayas.** Guayaquil, 26 Jun 1923, *Hitchcock 20141* (US, GH)***. C. psorothamnensis*** Stefanović et al. **U.S.A. California.** Imperial Co.: Near I-8 in sandy flat at mouth of In-Ko-Pah Gorge, Devil’s Canyon along Myer Creek, 17 Apr 1981, *Yatskievych 81–119* (ARIZ); San Diego Co.: Anza-Borrego Desert State Park, 3.2 air mi SW of Hwy 78/Split Mt. Rd., 3 Mar 2010, *Hendrickson* et al. *4502** (SD); Lois Neyenesch Folly private property, just NW of Anza-Borrego Desert State Park boundary, 1 mile W of Hwy S2 at Cranebrake Canyon Rd., 32.8972° N, 116.2422° W, 20 Mar 2005, *Nenow and Glacy 162** (SD); Anza-Borrego State Park, June Wash, 32.9775° N, 116.2475° W, 5 Mar 2005, *Angel 111* (SD); Anza-Borrego State Park, along Rd. S2, mile 51 and 52, 21 Apr 2013, *Stefanović SS-13-07** (TRTE, WLU). ***C. punana*** Costea & Stefanović: **ECUADOR, Guayas.** Isla Puná, Río Hondoto la Florida, 02^o^49’S, 80^o^01’W, 0 m, 7 Jun 1987, *Madsen 63850* (AAU); El Placer, 0–5 km on path toward Río Hondo, 02^o^48’S, 80^o^00’W, 8 Sep 1987, *Madsen 63936* (AAU). ***C. purpurata*** Philippi: **CANADA, Ontario.** Mississauga, grown in greenhouse at U of T, 13 Jul 2015, *Stefanović s.n.* (TRTE, WLU). **CHILE, Atacama.** Caldera, 10 m, 19 Feb 1939, *Beetle 26113* (G, S). ***C. purpusii*** Yunck. **MEXICO, Nuevo Leon**. Mpio. Galeana: Cerro el Viejo, Zaragoza, 1,930 m, 23 Sep 1993, *Hinton et al. 23503* (ARIZ, IEB). **Querétaro.** Mpio. Cadereyta: ca. 1Km NE of La Tinaja, 1,760 m, 16 Sep 2001, *Carranza & Silva 6242* (IEB). **San Luis Potosí.** Mpio. Guadalcázar, Cerro El Calvario, 22º36’N, 100º23’W, 1068 m, 31 Jul 2000, *Torres Colín 15864* (MEXU). **Tamaulipas.** Mpio. Altamira: 22 Km SE of Miquihuana, 2,500 m, 12 Aug 1941, *Stanford et al. 876* (NY). Mpio. Miquihuana: camino al Cañon del Soldado, 1900 m, 16 May 1986, *Martínez 1033* (MEXU). **C. *racemosa*** var. ***miniata*** (Mart.) Engelm.: Brazil, *Menezes et. al. 5100* (CTES); *Richon 7835* (S); *Arbo et al. 5100* (KEW); *Cordeiro et. al. 8211* (KEW). ***C. reflexa*** Roxb.: **INDIA, Sikkim.** Undated, *J.D. H. s.n.* (NY). **Punjab.** Kangra Bhadwar, 5 May 1933, *Walter Koelz 4397* (NY). **PAKISTAN, Punjab**. 5 Jan 1917, Mt. Tilla, *R. R. Stewart 734* (NY). ***C. rugosiceps*** Yunck.: **GUATEMALA, Quiché.** San Miguel Uspantan, Apr 1892, *Heyde & Lux 2912* (GH). **MEXICO, Oaxaca.** Sierra Madre del Sur, 20 Jun 1962, *Webster 11561* (GH). **Queretaro.** Jalpan, 21°28'11"N, 99°09'02"W, 3 Sep 1005, *Pérez-Calix 4603* (IEB, WLU). ***C. runyonii*** Yunck.: **U.S.A., Texas.** Hidalgo Co., off U.S. 83, 2 m E of Sullivan City on gravelly hill, 31 Mar 1941, *Lundell & Lundell 9827* (BRIT). ***C. salina*** Engelm.: **U.S.A.,** Arizona. Pima Co., Organ Pipe Cactus National Monument, 19 Jul 1989, *Felger & Fenn 89-241* (NY); Pinal Co., S end of reservoir Picacho Reservoir, 9 Apr 1996, *Hammond 10349* (NY); California: Alameda Co., N of Livermore, 31 Aug 1966, *Hoover 9950* (RSA); Nevada: Churchill Co., Lahontan Valley, 8 Sep 1998, *Tiehm & Bair 12744* (NY); Near spring SW of Sand Mtn, 24 Jul 1978, Williams *& Tiehm 78-233* (RSA). ***C. sidarum*** Liebm.: **COSTA RICA**. Puntarenas, 10**°00’00’’N, 84°42’00’’W, 100 m, 31 Jan 1993, *Hammel 18763* (F). MEXICO, Michoacan.** Hills between Río Tepalcatepec and Arteaga along the Hwy S, 350 m, 24 Feb 1965, *McVaugh 22526*(MICH). **Yucatán.** Chocholá, 4 km al E de la población de Chocholá, approx. 20^o^44’30”N, 89^o^47’20”W, 20-50 m, 7 Nov 2001, *Carnevali et al. 6425* (CICY)*.* **NICARAGUA, Managua.** Near Parque de Las Madres; ca 12^o^08’N, 86^o^16’W, 80 m, 30 Nov 1981, *Stevens 20950* (RSA). ***C. squamata*** Engelm.: **U.S.A., New Mexico.** Doña Ana Co., White Sands Missile Range, 3 mi E of Main Post, East Dry Lake Playa near Range Road 3 between LC 33 and C Station, 3900 ft, [no date], *Anderson 8057* (NMC); Collected on the Mesa W of the Organ Mountains, 22 Sep 1899, *Wooton s.n.* (NMC). **Texas.** El Paso, 10 Sep 1883, *Jones 4170* (RSA). ***C. strobilacea*** var. ***strobilacea*** Liebm.: **MEXICO, Jalisco.** Hillsides near Guadalajara, 10 Oct 1889, *Pringle 2472* (K, MEXU). **Morelos.** Along Hwy 115 D (Autopista toll road) between Cuautla and Cuernavaca, NW of Cuautla, 3.9 mi SE of junction with Hwy 95 D (between Cuernavaca and Mexico City), 18^o^59’N, 99^o^06’W, 1960 m, 24 Feb 1987, *Croat & Hannon 65757* (MO). ***C. suaveolens*** Seringe: **CHILE,** **Región de Arica y Parinacota.**, prov. Arica, valle de Codpa, 2000 m, 10 Apr 1974, *Castillo 98-74* (SGO). Valle de Azapa, 18°30’S, 69°45’W, 840 m, 22 Mar 1987, *Matthei & Rodríguez 350*, 376 (CONC). **U.S.A., California.** Humboldt Co., Myers Ranch, S Fork of Eel River 8 mi above the mouth, 200 ft, 29 Sep 1918, *Tracy 5113* (JEPS, UC1); Kern Co., Rosedale, 30 Sep 1894, *Abrams 458* (RSA). ***C. subinclusa*** Durand & Hilgard: **U.S.A., California.** Kern Co., Kernville, 2650 ft, 25 Sep 1970, *Howell 47416* (NY); Riverside Co., San Gorgonio wash at S. P. RR, San Gorgonio Pass, 2100 ft, 1 Dec 1933, *Wheeler 2284* (RSA); San Diego Co., chaparral, 4 mi W of Hwy 94 on road to Otay Reservoir, N base of San Ysidro Mountains, 800 ft, 20 Aug 1952, *Munz & Balls 17942* (NY); San Luis Obispo Co., Rinconada district, below Santa margarita and Pégo, 15 Sep 1946, *Hoover 6401* (RSA). ***C. suksdorfii*** Yunck.: **U.S.A., California.** Mariposa Co. Yosemite National Park, NAD27 Zone 11 280152E 4189262N, 8710 feet, 20 Jul 2004, *Colwell AC 04-159* (UC1); Tuolumne Co., Mineral spring near John Muir Trail in Lyell Canyon 1.4 km E of Rafferty Creek, Yosemite National Park UTM, Zone 11 297096E 4193313N, 2670 m, 8 Sep 2005, *Colwell et al. AC05-233* (UC1); Siskiyou Co., S side of Preston Peak, Rattlesnake Meadow, 25 Aug 1963, *Wheeler 8269* (RSA). ***C. tasmanica*** Engelm.: **AUSTRALIA, Victoria.** Volcanic Plain, By Causeway between Lke Corangamite and Lake Martin, W of Berrybank- Cundare Road, 38^o^06’S, 142^o^33’E, Towards Lake Corangamite on N side of causeway, 29 Jan 1991, *Walsh 3045* (MEL). ***C. timida*** Costea and Stefanović **MEXICO.** **Hidalgo**. Along Hwy 85 between Tamazunchale and Zimapàn, 40 Km N of Zimapan, 10 Oct 1985, *Spellenberg et al. 8359** (NMC, MEXU). **Puebla**. Tehuacán, [no date], *Rose s.n.** (NY). **Querétaro**. Mpio. Colón, SE of Cerro Zamorano, 3 Km N of Los Trigos, 2,750 m, 30 Sep 2002, *Rzedowski 54044** (IEB, WLU); **Veracruz**. Mpio. Maltrata: Maltrata, Jan 1883, *Kerber 248* (UPS); El Puerto above Orizaba, 2,316 m, 5 Sep 1944, *Sharp 44637* (GH). ***C. tinctoria*** var. ***aurea*** (Liebm.) Costea: **MEXICO, San Luis Potosi.** Unknown locality, 12-16 Sep 1902, *Palmer 89* (S). ***C. tinctoria*** var. ***floribunda*** (Kunth) Costea: **MEXICO, Veracruz.** Maltrata, Jan 1883, *Kerber s.n.* (KEW). ***C. tinctoria*** var. ***tinctoria*** Martius: **MEXICO, Hidalgo.** 4 km al SE de Tolcayuca, 21 Nov 1978, *Ortega s.n.* (MEXU). **Mexico.** 1 km al N de San Juan Citlatepec, mpio. De Zumpango, 2240 m, *Rzedowski ME-22* (MEXU). **Puebla.** Cuapiaxtla, cerca de Tepeaca, 2000 m, 6 Aug 1965, *Rzedowski 20380* (MEXU); 1 km al SE de San Hipól.- to Xochiltenango, 11 Sep 1961, *Sousa s.n.* (MEXU). **San Luis Potosi.** Unknown locality, 1877, *Schaffner 781* (MEXU). ***C. tolteca*: MEXICO, Guanajuato.** Xichú, 25 July 1997, *E. Carranza & R. M. García 5331* (IEB, WLU); **Queretaro.** Cadereyta, 20°40'16"N 99°31'32"W., 29 Sep 2007, *Zamudio & Guevara 13882* (IEB, WLU); El Marqués. 28 July 1999, *G. Ocampo 856* (IEB, WLU). ***C. tuberculata*** Brandegee: **MEXICO, Baja California Sur.** Hwy. 1, KP 20, 12 mi NE Villa Insurgentes El. 250’, 7 Sep 1983, *Donahue 73168* (RSA); Arroyo de Santa Agueda, SW of Santa Rosalía on road to Santa Agueda, 27^o^15’N, 112^o^24’W, 3 Oct 1951, *Carter & Kellogg 3085* (NY, RSA). **Sonora.** Bavispe, 3850 ft, 19 Oct 1890, *Lumholtz 179* (GH). ***C. umbellata*** Kunth: **GUIANA,** Georgetown. Seacoast E of sea wall, 29 Oct 1919, *Hitchcock 16564* (S). **MEXICO, Guerrero.** Coahuayutla de Guerrero, La Corva, 5.46 km al N, 18^o^32’8”N, 101^o^50’28”W, 20 Oct 1999, *Soto 17626* (MEXU). **Jalisco.** ca. 4.5 mi from Zacoalco beside road to Acatlán, 1350-1375 m, *Dieterle 3471* (MICH). **Oaxaca.** Cuicatlán, San Juan Bautista Cuitcatlan, 10.2 km al NW del Chilar, 17^o^47’21”N, 96^o^59’31”W, 665 m, 1 Oct 2002, *Soto 24009* (MEXU). **Puebla.** Calcareous hills near Tehuacan, 5500 ft, 24 Dec 1895, *Pringle 6297* (S); Tehuacán, 9 Aug 1938, *Kenoyer A307* (F). **U.S.A., New Mexico.** Doña Ana Co., NW of Doña Ana Community College on ground next to corral, Las Cruces, 15 Oct 1990, *Silversmith s.n.* (NMC). ***C. umbrosa*** Beyr. ex Hook.: **CANADA, Manitoba.** Distr. De Saint-Boniface. Rivière Rouge, ecorre de la Rouge à La Fourche, 21 Aug 1960, *Boivin 13852* (DAO); Otterbourne, 4 Aug 1954, *Bernard 54/349* (QFA); Sans Souci, 21 Jul 1956, *Bernard 56/5473* (QFA); Winnepeg, sent to G. Knowles, Field Husbandry by M. R. Mackenzie 419 Kingston Crescent, 1950, *Mackenzie s.n.* (DAO); 13 Aug 1954, *Bernard 497* (DAO). **Saskatchewan.** Little Manitou Lake, N side of the lake, 20 Aug 1992, *Hudson 5082* (USAS). **U.S.A., Colorado.** Dome Rock in Platte Canyon, 7000 ft, 8 Aug 1878, *Jones 571* (RSA). ***C. vandevenderi*** Costea & Stefanović: **MEXICO, Sonora.** Álamos, El Palmarito, 27°03'04"N 108°45'51"W, 1 Oct 2006, *Van Devender 2006-983* (WLU); Yécora, 28°22'40"N 109°09'W, 20 Sep 1998, *Van Devender 98-1434* (WLU). ***C. victoriana*** Yunck.: **AUSTRALIA, Northern Territory.** 6 mi NW of Mt. Swan Station, ca. 22^o^36’S, 135^o^02’E, 11 Mar 1953, *Perry 3329* (CANB). **South Australia.** Lake Eyre Region, between Hough’s Dam and Chapman’s Creek Tank, Dulkaninna Station, 29^o^04’23”S, 138^o^37’28”E, 9 Apr 1997, *Smyth 261* (CANB). **Western Australia.** Rear of Bullgarra cell, Karratha, Jul 1987, *Glennon 379* (CANB). ***C. volcanica*** Costea & I. García: **MEXICO, Jalisco.** Tonila, Volcán Nevado de Colima, 3100 m, *Zamudio 4274* (MICH). **Puebla.** Azumbilla, Puerto del Aire, carr. A Orizaba, 3 Jan 2002, *Tenorio 21748* (MEXU); Caltepec, El Ojo de Agua, al E de Caltepec, 18^o^4’N 97^o^25’W, 1250 m, 28 Dec 2001, *Tenorio & Kelly 21688* (MEXU). ***C. warnerii*** Yunck.: **U.S.A., New Mexico.** Sierra Co., Pedro Armendaris Grant, 15.6 mi N of Engle, E of Red Lake, 4800 feet, 24 Sep 1998, *Peterson 98-699* (NMC). **Utah.** Millard Co., Vicinity of Flowell, 15 mi W of Fillmore, 10 Sep 1957, *Warner s.n.* (UC, isotype). ***C. werdermanii*** Hunz.: **CHILE. [**Unknown locality, unknown date], *Reiche s.n.* (SGO). ***C. woodsonii*** Yunck.: **MEXICO, Hidalgo.** 3.5 km NW of Zimapan on road to La Purisma, 1800 m, 20^o^46’N 99^o^25’W, 9 Oct 1985, *Spellenberg 8334* (NMC); along Mexico Highway 85 between Tamazinchale and Zimapan, 90 km S of border with San Luis Potosi, 40 km N of Zimapan, 10 Oct 1985, *Spellenberg et al. 8359* (NMC). **PANAMA, Chiriqui.** Lava field and slopes between Volcan de Chiriqui and Cerro Aquacate, 6500-7200 ft, 16 Jan 1971, *Wilbur et al. 13316* (MO); Road to top of El Baru from Boquete, E side of Baru, 7600-9000 ft, 17 Mar 1979, *D’Arcy et al. 12622* (MO). ***C. xanthochortos*** Mart. ex Engelm. var. ***carinata*** (Yunck.) Yunck.: **PARAGUAY, Cordillera.** Cerrado forest, 25^o^07’S, 57^o^19’W, 1 Jun 1993, *Zardini & Guerrero 35961* (MO, WLU). ***C. yucatana*** Yunck.: **MEXICO, Chiapas**. Mpio of Tenejapa: Paraje Tenejapa, 2700 m, 7 Nov 1971, *Breedlove & Sith 22017* (MEXU). **Hidalgo**. Presa Jaramillo, 4 Km N of Pachuca, 2800 m, 29 Jul 1978, *Rzedowski 25758* (G, MEXU). **Puebla**. Mpio. Tepeyahualco, Laguna Salado, 6 Km N of Laguna Alchichica, 19°28'N, 97°25'W, 2300 m, 20 Feb 1980, *Nee and Taylor 29575* (F, MO, XAL). **Yucatán**. Valladolid, 22 m, 2 Jul 1932, *Steere 1695* (F, NY)
