# Supplementary material for: Inflorescences of Cuscuta (Convolvulaceae): Diversity, evolution and relationships with breeding systems and fruit dehiscence modes
Source: PLoS One. 2023 May 19;18(5):e0286100. doi: 10.1371/journal.pone.0286100 (PMC10198556; doi:10.1371/journal.pone.0286100)
Supplement: S2 Appendix — Infrageneric classification from [4]. Pollen-ovule ratio data from [15] except were indicated by an asterisk (*). Modes of dehiscence from [19]. IN = indehiscent; IrB = irregular type B; IrA = irregular type A; DE = dehiscent. (DOCX) [file pone.0286100.s005.docx]

**Appendix S2**. Inflorescence, flower, pollen-ovule ratios and fruit characters. Infrageneric classification from [4]. Pollen-ovule ratio data from [15] except were indicated by an asterisk (*). Modes of dehiscence from [19]. IN = indehiscent; IrB = irregular type B; IrA = irregular type A; DE = dehiscent.

| Taxon | Pedicel length (mm) | Total axes length (mm) | Corolla tube length (mm) | Corolla diameter (mm) | Pollen-ovule ratios | Fruit width (mm) | Fruit length (mm) | Modes of dehiscence |
| --- | --- | --- | --- | --- | --- | --- | --- | --- |
| **SUBG. GRAMMICA**  **Sect. Californicae** |  | | | | | | | |
| *Cuscuta brachycalyx* | 1.83 | 2.89 | 2.42 | 4.11 | 987.5 | 2.2 | 1.5 | IN |
| *C. occidentalis* | 1.05 | 1.06 | 2.11 | 4.87 | 1400.25 | 2.41 | 1.73 | IN+IrB |
| *C. californica* var. *californica* | 1.58 | 1.02 | 2.24 | 4.79 | 1689 | 2.84 | 2.5 | IN+IrB |
| *C. californica* var. *papillosa* | 0.85 | 0.9 | 2.02 | 6.6 | 746* | 1.8 | 2.3 | IN+IrB |
| *C. salina* | 1.58 | 2.85 | 2.14 | 3.36 | 792.22 | 1.33 | 1.7 | IN |
| *C. pacifica* | 0.51 | 1.52 | 2.19 | 4.01 | 406.5 | 1.52 | 2.25 | IN |
| *C. suksdorfii* | 0.54 | 0.72 | 1.11 | 2.41 | 186.06 | 2.09 | 1.82 | IN+IrB |
| *C. subinclusa* | 0.91 | 3.23 | 3.72 | 4.17 | 989.79 | 2.47 | 3.88 | IN |
| *C. howelliana* | 0.21 | 0.42 | 1.43 | 1.55 | 985.5 | 2.02 | 1.6 | IN |
| *C. decipiens* | 1.15 | 1.69 | 2.5 | 2.93 | 1274 | 1.65 | 2.3 | IN |
| **Sect. Cleistogrammica** |  | | | | | | | |
| *C. obtusiflora* var. *glandulosa* | 0.84 | 2.09 | 3.06 | 3.99 | 803 | 2.65 | 1.4 | IN+IrB |
| *C. obtusiflora* var. *obtusiflora* | 0.87 | 1.44 | 1.92 | 4.08 | 228 | 2.34 | 1.81 | IN+IrB |
| *C. australis* var*. tinei* | 0.56 | 1.24 | 1.88 | 4.01 | 260 | 2.98 | 2.2 | IN+IrB |
| *C. australis* var. *australis* | 0.8 | 1.1 | 1.35 | 2.43 | 311.5 | 3.6 | 2.6 | IN+IrB |
| *C. campestris* | 1.29 | 2.22 | 1.18 | 3.23 | 284.58 | 3.44 | 2.43 | IN+IrB |
| *C. pentagona* | 0.88 | 2.17 | 1.07 | 2.32 | 409.5 | 2.2 | 1.55 | IN+IrB |
| *C. harperi* | 0.61 | 1.67 | 0.65 | 1.41 | 229.5 | 1.61 | 1.01 | IN+IrB |
| *C. plattensis* | 1.62 | 1.82 | 2.89 | 3.75 | 841.5 | 3.62 | 2.8 | IN |
| *C. runyonii* | 1.32 | 2.32 | 2.27 | 3.67 | 1046.5 | 2.6 | 2 | IN |
| *C. glabrior* | 1.54 | 2.28 | 1.94 | 3.56 | 972.13 | 2.8 | 2.3 | IN |
| *C. polygonorum* | 0.52 | 1.16 | 1.1 | 2.75 | 168.5* | 3.62 | 2.37 | IN |
| **Sect. Racemosae** |  | | | | | | | |
| *C. micrantha* | 1.68 | 2.4 | 1.34 | 3.05 | 2025.33 | 1.54 | 1.54 | IN |
| *C. xanthochortos* var. *carinata* | 0.42 | 3.9 | 1.1 | 4.02 | 982.63 | 2.36 | 2.07 | DE+IrA |
| *C. corniculata* | 1.41 | 2.04 | 1.78 | 4.03 | 438.63 | 2.44 | 2.1 | IrA |
| *C. suaveolens* | 3.78 | 7.11 | 2.06 | 4.28 | 508 | 2.6 | 2.2 | IN |
| *C. werdermannii* | 4.21 | 7.09 | 2.69 | 4.11 | 405.5 | 2.7 | 2.48 | IN |
| *C. parviflora* var. *elongata* | 2.89 | 4.01 | 1.06 | 2.07 | 916.67 | 2.19 | 1.85 | IN |
| *C. racemosa* var. *miniata* | 1.47 | 4.76 | 2.44 | 3.11 | 222.5 | 1.82 | 1.45 | IN |
| *C. platyloba* | 1.47 | 2.44 | 1.47 | 3.22 | 517.25 | 2.72 | 2.62 | IN |
| *C. incurvata* | 0.92 | 5.17 | 1.42 | 3.78 | 369.58 | 2.49 | 2.2 | DE+IrA |
| **Sect. Oxycarpae** |  | | | | | | | |
| *C. cuspidata* | 0.59 | 7.72 | 1.75 | 3.61 | 1164.25 | 3.26 | 2.18 | IN |
| *C. squamata* | 0.54 | 1.15 | 2.55 | 3.17 | 1801.63 | 2.04 | 3.09 | IN |
| *C. compacta* | 0.33 | 5.26 | 3.35 | 3.33 | 688.25 | 4.56 | 4.95 | IN+IrB |
| *C. rostrata* | 1.42 | 4.57 | 4.14 | 4.61 | 1132.5 | 4.07 | 4.92 | IN+IrB |
| *C. gronovii* var. *gronovii* | 1.62 | 5.39 | 2.67 | 4.14 | 1290.5 | 5.41 | 4.82 | IN+IrB |
| *C. gronovii* var. *latiflora* | 1.19 | 2.05 | 1.32 | 3.1 | 669.5 | 2.98 | 2.64 | IN+IrB |
| *C. cephalanthi* | 1.16 | 4.2 | 1.75 | 2.25 | 261.5 | 3.5 | 2.67 | IN+IrB |
| *C. umbrosa* | 1.49 | 4.03 | 2.66 | 2.06 | 371.25 | 5.18 | 5.22 | IN+IrB |
| *C. glomerata* | 0.1 | 0.1 | 3.91 | 4.42 | 781.25 | 2.81 | 3.08 | IN+IrB |
| **Sect. Denticulatae** |  | | | | | | | |
| *C. denticulata* | 0.47 | 0.93 | 1.48 | 1.62 | 1011.75 | 1.27 | 1.69 | IN |
| *C. nevadensis* | 1.34 | 0.3 | 1.8 | 2.49 | 727.42 | 1.23 | 1.53 | IN |
| *C. psorothamnensis* | 0.89 | 0.4 | 1.91 | 2.63 | 637* | 1.25 | 1.57 | IN |
| **Sect. Partitae** |  | | | | | | | |
| *C. haughtii* | 2.13 | 5.24 | 1.46 | 3.31 | 405.5 | 2.11 | 1.41 | IN |
| *C. partita* | 1.72 | 5.21 | 2.46 | 3.86 | 453.83 | 2.45 | 1.56 | DE+IrA |
| *C. longiloba* | 1.59 | 4.33 | 2.7 | 3.21 | 361.21 | 2.14 | 1.75 | IN |
| **Sect. Lobostigmae** |  | | | | | | | |
| *C. lindsayi* | 1.78 | 8.24 | 4.21 | 3.89 | 3114.42 | 4.1 | 3.4 | DE |
| *C. tinctoria* var. *tinctoria* | 1.69 | 7.34 | 2.49 | 5.23 | 1598 | 2.8 | 2.46 | DE |
| *C. tinctoria* var. *aurea* | 1.22 | 6.11 | 1.87 | 4.66 | 1481 | 2.6 | 2.2 | DE |
| *C. tinctoria* var. *floribunda* | 0.85 | 4.28 | 2.81 | 5.22 | 2407.27 | 3.2 | 2.52 | DE |
| *C. mitriformis* | 1.35 | 2.42 | 1.9 | 4.24 | 383.58 | 4.11 | 3.92 | DE |
| *C. jalapensis* | 1.46 | 7.36 | 2.73 | 5.36 | 1268 | 4.7 | 4.6 | DE |
| *C. rugosiceps* | 1.17 | 2.34 | 2.36 | 5.81 | 949 | 4.2 | 3.9 | DE |
| *C. woodsoni* | 0.46 | 1.68 | 3.69 | 4.23 | 1069.5 | 5.8 | 4.22 | DE |
| *C. volcanica* | 1.38 | 1.83 | 4.2 | 7.46 | 4197 | 5.5 | 4.33 | DE |
| *C. purpusii* | 1.42 | 6.55 | 2.68 | 4.21 | 1285.75 | 3.02 | 2.72 | DE |
| *C. victoriana* | 1.58 | 0.42 | 0.91 | 2.6 | 118.92 | 3.71 | 1.74 | IN |
| *C. tasmanica* | 7.1 | 0.1 | 2.47 | 4.73 | 930.08 | 3.32 | 3.08 | IN |
| *C. cotijana* | 1.54 | 5.01 | 4.84 | 5.57 | 2957.5 | 3.61 | 3.2 | DE |
| *C. timida* | 0.76 | 0.88 | 3.78 | 4.97 | 2249.5* | 3.48 | 2.93 | DE |
| *C. tolteca* | 0.39 | 1.12 | 3.2 | 4.98 | 3655.5* | 3.71 | 2.82 | DE |
| *C. iguanella* | 0.32 | 0.92 | 3.01 | 4.1 | 1622 | 3.9 | 2.1 | DE |
| **Sect. Grammica** |  | | | | | | | |
| *C. potosina* | 0.67 | 0.94 | 1.27 | 2.76 | 242.25 | 0.9 | 1.3 | DE |
| *C. azteca* | 0.83 | 1.48 | 1.1 | 2.26 | 739.75 | 2.11 | 1.3 | DE |
| *C. yucatana* | 1.16 | 1.79 | 1.12 | 2.56 | 203.3 | 2.33 | 1.4 | IN+IrA |
| *C. chinensis* var. *applanata* | 1.05 | 2.3 | 1.98 | 3.84 | 538.25 | 2.33 | 1.52 | DE |
| *C. chinensis* var. *chinensis* | 1.07 | 1.99 | 2.1 | 4.21 | 586.25 | 2.22 | 1.44 | DE |
| **Sect. Obtusilobae** |  | | | | | | | |
| *C. macrocephala* | 2.1 | 2.28 | 4.3 | 4.21 | 4049.75 | 3.3 | 2.6 | DE |
| *C. cozumeliensis* | 1.4 | 3.13 | 3.29 | 2.42 | 875.63 | 1.83 | 1.48 | DE |
| *C. globulosa* | 1.86 | 11 | 3.1 | 2.26 | 552.7* | 2.52 | 1.62 | DE |
| *C. americana* | 0.92 | 5.38 | 2.68 | 1.57 | 275.05 | 2.2 | 1.99 | DE |
| **Sect. Prismaticae** |  |  |  |  |  |  |  |  |
| *C. corymbosa* var. *stylosa* | 1.48 | 4.76 | 3.14 | 2.2 | 1545.76 | 1.72 | 1.31 | DE |
| *C. corymbosa* var. *grandiflora* | 2.46 | 4.02 | 5.14 | 1.8 | 1624.44 | 1.88 | 1.51 | DE |
| *C. prismatica* | 0.91 | 3.1 | 5.2 | 2.12 | 552.75 | 1.54 | 1.33 | DE |
| **Sect. Ceratophorae** |  | | | | | | | |
| *C. chapalana* | 1.22 | 5.51 | 3.25 | 3.13 | 2331.45 | 1.76 | 1.55 | DE |
| *C. mexicana* | 1.72 | 4.83 | 2.13 | 4.11 | 1432 | 3.36 | 1.5 | DE |
| *C. strobilacea* var. *strobilacea* | 0.9 | 1.6 | 3.14 | 5.32 | 1741.75 | 3.3 | 2.6 | DE |
| *C. erosa* | 2.7 | 7.8 | 2.01 | 4.55 | 1184.63 | 2.5 | 1.85 | DE |
| *C. boldinghii* | 0.82 | 2.18 | 1.88 | 3.66 | 409.13 | 2.4 | 1.7 | DE |
| *C. costaricensis* | 1.51 | 2.05 | 3.49 | 3.73 | 881.08 | 3.2 | 2.1 | DE |
| *C. bonafortunae* | 0.73 | 2.37 | 1.09 | 2.84 | 1123* | 2.3 | 1.8 | DE |
| *C. odontolepis* | 1.04 | 2.13 | 2.67 | 4.46 | 1552.5 | 2.25 | 1.9 | DE |
| **Sect. Umbellatae** |  | | | | | | | |
| *C. legitima* | 3.37 | 6.4 | 1.71 | 5.65 | 1296.17 | 2.3 | 1.65 | DE |
| *C. tuberculata* | 2.24 | 2.26 | 1.91 | 1.67 | 916.13 | 2.01 | 1.51 | DE |
| *C. umbellata* | 4.52 | 6.3 | 1.07 | 3.2 | 427.5 | 2.09 | 1.62 | IN+DE+IrA |
| *C. desmouliniana* | 1.37 | 4.6 | 1.2 | 3.3 | 677 | 2.05 | 1.52 | DE |
| *C. hyalina* | 2.1 | 4.3 | 1.64 | 4.48 | 362.5 | 2.01 | 1.59 | DE |
| *C. acuta* | 1.56 | 1.72 | 1.67 | 4.82 | 245.6 | 2.5 | 2 | IN+IrA |
| *C. leptantha* | 3.74 | 2.78 | 2.13 | 4.12 | 778.54 | 1.8 | 1.5 | DE |
| *C. polyanthemos* | 6.28 | 3.32 | 5.26 | 4.17 | 1178.75 | 2.25 | 1.2 | DE |
| *C. liliputana* | 3.22 | 2.86 | 1.65 | 4.34 | 405.25* | 2.19 | 1.1 | DE |
| *C. membranacea* | 2.11 | 3.12 | 1.41 | 4.11 | 281.25* | 2.62 | 1.96 | DE |
| **Sect. Indecorae** |  | | | | | | | |
| *C. indecora* var. *indecora* | 4.11 | 4.24 | 1.86 | 3.66 | 943 | 3.2 | 2.7 | IN |
| *C. coryli* | 2.2 | 2.22 | 1.55 | 2.23 | 156.5 | 4.23 | 2.77 | IN+IrB |
| *C. warnerii* | 0.47 | 0.46 | 1.1 | 1.19 | 140.25 | 3.3 | 2.65 | IN |
| **Sect. Gracillimae** |  |  |  |  |  |  |  |  |
| *C. deltoidea* | 4.14 | 5.32 | 1.06 | 1.79 | 629 | 1.2 | 0.9 | DE |
| *C. vandevenderi* | 1.86 | 4.79 | 1.08 | 2.27 | 372.5 | 1.8 | 1.1 | IN |
| *C. sidarum* | 2.72 | 5.1 | 1.64 | 3.37 | 661.38 | 1.4 | 1.2 | DE |
| *C. colombiana* | 2.89 | 5.98 | 1.57 | 2.98 | 453* | 1.2 | 0.9 | DE+IrA |
| *C. gracillima* | 2.53 | 4.73 | 1.25 | 3.23 | 2243 | 1.5 | 1.3 | DE |
| *C. punana* | 2.33 | 4.36 | 1.29 | 2.46 | 457 | 1.82 | 1.23 | DE+IrA |
| *C. macvaughii* | 2.21 | 4.51 | 1.91 | 3.72 | 1611.75 | 1.63 | 1.23 | IN |
| **Sect. Subulatae** |  | | | | | | | |
| *C. cockerellii* | 0.34 | 1.62 | 3.41 | 3.33 | 1534 | 2.57 | 2.2 | DE |
| *C. chilensis* | 1.35 | 2.45 | 4.79 | 5.21 | 1654.44 | 4.06 | 3.21 | DE |
| *C. odorata* | 0.89 | 2.38 | 2.69 | 7.31 | 2017.75 | 2.87 | 2.1 | DE |
| *C. purpurata* | 0.86 | 3.15 | 2.38 | 7.35 | 1705.5 | 2.57 | 1.52 | DE |
| *C. foetida* var*. foetida* | 2.96 | 2.22 | 3.89 | 5.89 | 708.08 | 2.8 | 2 | DE |
| *C. foetida* var. *pycnantha* | 1.12 | 3.2 | 5.67 | 6.11 | 708.08 | 2.9 | 2.1 | DE |
| *C. paitana* | 2.39 | 2.36 | 3.56 | 5.62 | 1039.75 | 2.6 | 1.8 | DE |
| *C. globiflora* | 0.71 | 1.46 | 3.39 | 2.75 | 886.5 | 3.56 | 1.97 | DE |
| *C. grandiflora* | 2.89 | 0.42 | 2.3 | 7.12 | 358.69 | 3.6 | 1.1 | DE |
| *C. parodiana* | 1.98 | 5.94 | 3.75 | 4.74 | 1327.63 | 2.9 | 2.1 | DE |
| *C. kilimanjari* | 1.51 | 5.8 | 2.86 | 5.47 | 1060.5 | 3.1 | 2.2 | DE |
| *C. cristata* | 1.45 | 1.47 | 2.2 | 3.62 | 577.25 | 3.11 | 2.11 | IN+IrA |
| *C. argentinana* | 2.76 | 2.63 | 1.64 | 6.22 | 1575.75 | 3.27 | 2.12 | DE |
| *C. friesii* | 4.44 | 3.95 | 1.58 | 6.11 | 1021.71 | 4.45 | 2.2 | DE |
| *C. microstyla* | 0.64 | 1.66 | 2.17 | 4.28 | 1295.75 | 2.1 | 1.3 | IN |
| **SUBG. PACHYSTIGMA** |  | | | | | | | |
| *C. natalensis* | 2.6 | 6.11 | 2.32 | 5.41 | 1689.75 | 2.3 | 1.4 | DE |
| *C. nitida* | 2.9 | 8.74 | 2.21 | 4.96 | 1452.13 | 2.06 | 1.3 | DE |
| *C. angulata* | 3.8 | 11.4 | 1.6 | 4.48 | 1089.25 | 1.99 | 1.27 | DE |
| *C. africana* | 2.2 | 9.1 | 1.66 | 5.32 | 730.38 | 2.2 | 1.24 | DE |
| **SUBG. CUSCUTA** |  | | | | | | | |
| *C. europaea* | 0.23 | 0.1 | 2.11 | 2.21 | 232.6 | 2.79 | 2.2 | DE |
| *C. epilinum* | 0.26 | 0.1 | 1.33 | 2.56 | 277.91 | 2.85 | 2.3 | DE |
| *C. approximata* | 0.21 | 0.1 | 0.98 | 3.34 | 120.17 | 2.22 | 1.72 | DE |
| *C. epithymum* | 0.18 | 0.1 | 1.23 | 3.53 | 1019.25 | 1.72 | 1.53 | DE |
| *C. planiflora* | 0.2 | 0.1 | 1.47 | 2.1 | 231.5 | 1.72 | 1.34 | DE |
| **SUBGENUS MONOGYNELLA** |  | | | | | | | |
| *C. exaltata* | 1.25 | 20.88 | 2.42 | 2.18 | 454 | 5.47 | 5.88 | DE |
| *C. lehmanniana* | 1.09 | 20.04 | 3.52 | 5.09 | 2341.56 | 4.51 | 6 | DE |
| *C. monogyna* | 0.52 | 15.84 | 1.92 | 1.67 | 366.33 | 4.6 | 4.9 | DE |
| *C. japonica* | 1.43 | 16.97 | 2.65 | 5.22 | 856.4 | 4.45 | 5.14 | DE |
| *C. reflexa* | 1.49 | 18.11 | 4.16 | 4.52 | 2006.38 | 5.17 | 6.28 | DE |
| *C. cassythoides* | 0.58 | 17.1 | 1.85 | 4.76 | 484.67 | 6.1 | 4.99 | DE |
| *C. lupuliformis* | 1.66 | 16.96 | 2.58 | 2.705 | 167.92 | 3.78 | 4.2 | DE |
